# Toward the Clinical Translation of Implantable Brain–Computer Interfaces for Motor Impairment: Research Trends and Outcome Measures

**DOI:** 10.1002/advs.202501912

**Published:** 2025-07-23

**Authors:** Esmee Dohle, Eleanor Swanson, Luka Jovanovic, Suraya Yusuf, Lucy Thompson, Hugo Layard Horsfall, William Muirhead, Luke Bashford, Jamie Brannigan

**Affiliations:** ^1^ Oxford University Hospitals John Radcliffe Hospital Oxford OX3 9DU UK; ^2^ Utrecht University Medical Centre Utrecht 3584 CX Netherlands; ^3^ East and North Hertfordshire NHS Trust Lister Hospital Stevenage SG1 4AB UK; ^4^ Faculty of Medicine Imperial College London London SW7 5NH UK; ^5^ Department of Neurosurgery National Hospital for Neurology and Neurosurgery London WC1N 3BG UK; ^6^ The Francis Crick Institute London NW1 1AT UK; ^7^ Institute of Neurology University College London London WC1N 3BG UK; ^8^ Bioscience Institute, Faculty of Medical Sciences Newcastle University Newcastle upon Tyne NE1 7RU UK; ^9^ Department of Neurosurgery University of Colorado Denver CO 80045 USA; ^10^ Department of Medical Physics and Biomedical Engineering University College London London WC1E 6BT UK

**Keywords:** BCIs, brain–computer interfaces (BCIs), outcome measures, trends

## Abstract

Implantable brain–computer interfaces (iBCIs) decode neural signals to control external effectors, offering potential to restore function in individuals with severe motor impairments, such as loss of limb function or speech. This systematic review examines the evolution of iBCI research and key bottlenecks to clinical translation, particularly the absence of standardized, clinically meaningful outcome measures. A comprehensive search of MEDLINE, Embase, and CINAHL identifies 112 studies, nearly half (49.1%) published since 2020. Eighty unique iBCI participants were identified, providing the most up‐to‐date estimate of global users. Research remains concentrated in the United States (83%), with growing contributions from Europe, China, and Australia. Electrocorticography (ECoG)‐based devices increasingly emerge alongside micro‐electrode arrays. iBCI devices are now being used to control a broader range of effectors, including robotic prosthetics and digital technologies. Although most (69.6%) studies reported outcome measures prospectively, these primarily related to decoding (69.6%) and task performance (62.5%), with only 17.9% assessing clinical outcomes. When cassessed, clinical outcomes were highly heterogeneous due to varied approaches across target populations. iBCIs show potential to restore functional independence at scale. However, challenges remain around cross‐subject generalization, scalable implantation, and outcome standardization. Novel measures should be developed collaboratively with engineers, clinicians, and individuals with lived experience of motor impairment.

## Graphical abstract

Implantable brain–computer interfaces (iBCIs) are rapidly approaching clinical translation. This review maps key trends in the development of iBCIs and highlights the lack of standardised clinical outcome measures. It discusses a proposed framework for the selection of outcome measures in clinical trials and introduces a new international registry of iBCI clinical trial participants to support collaboration.

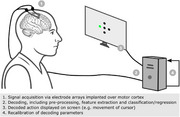

## 1 Introduction

Millions of individuals worldwide suffer from neurological conditions which impair the transmission of motor intentions from the brain to muscles, preventing them from independently performing activities of daily living (ADL). The disease burden of such conditions, which include stroke, amyotrophic lateral sclerosis (ALS), and spinal cord injury (SCI), is rapidly increasing, with neurological diseases now the leading cause of ill health worldwide.^[^ ^1^ ^]^ Furthermore, most individuals with SCI sustain their injury before the age of 50, resulting in decades with increased dependence on care and reduced quality of life.^[^ ^2^, ^3^, ^4^ ^]^

**Figure 1:**
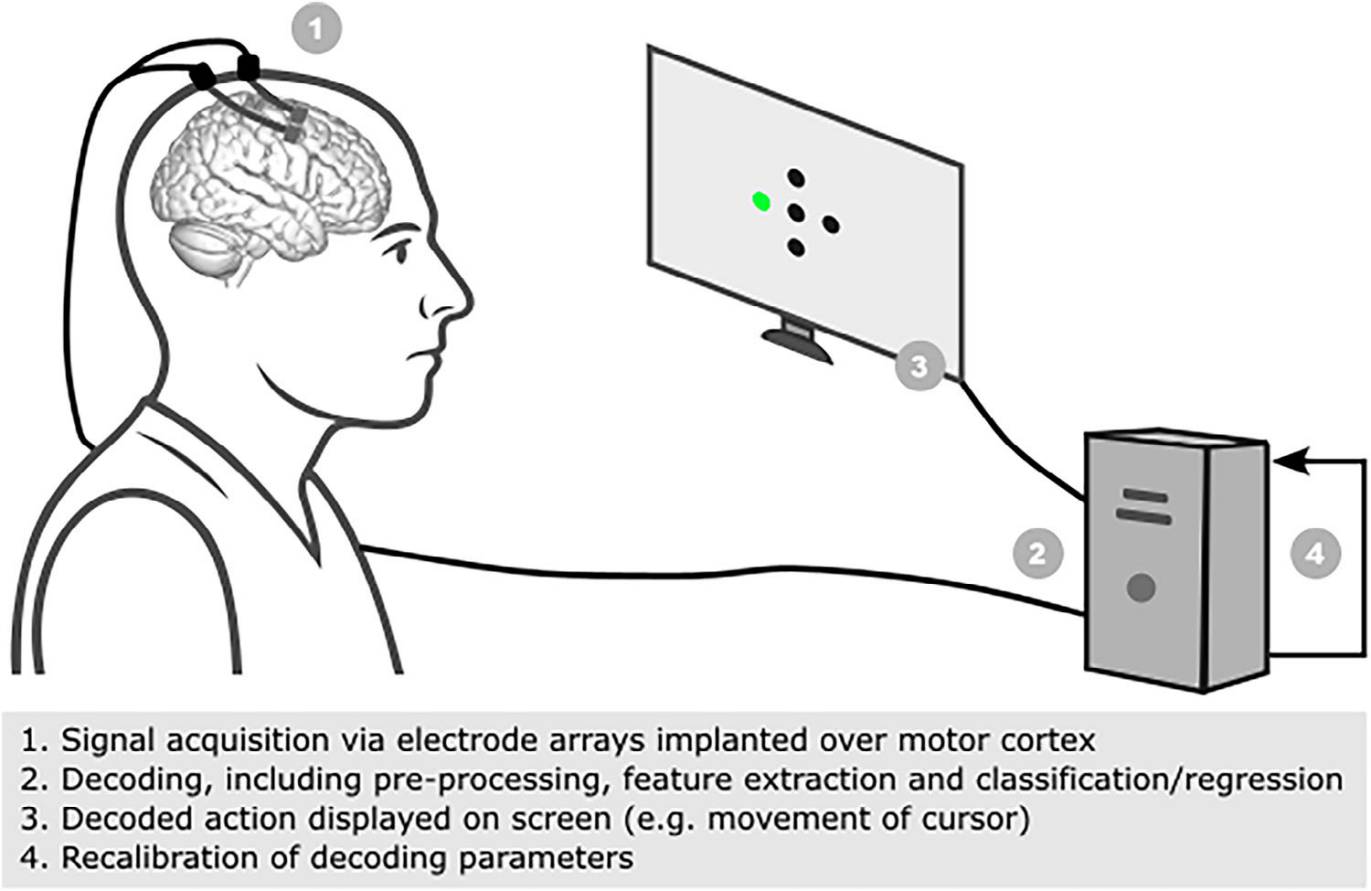
Example of experimental setup of an implantable brain–computer interface with a digital effector.

In recent decades, brain–computer interfaces (BCIs) have emerged as a transformative technology to restore function by decoding preserved neural signals and transmitting this activity via computers to effector devices, enabling the performance of desired actions. These prosthetic systems function by recording neural signals, decoding the signals into desired output actions, and executing these actions via an effector in the environment. Various systems have been investigated to accomplish each of these steps. Sensors may be implanted intracranially, such as micro‐electrode arrays (MEAs), surface ECoG arrays, or endovascular stent arrays. Alternatively, sensing may be non‐implantable, using wearable device techniques like scalp electroencephalography (EEG) and functional near‐infrared spectroscopy (fNIRS).^[^ ^5^ ^]^ Decoding of neural signals may be performed using a variety of neural signal processing techniques. Outputs can drive a range of effectors, including robotic prosthetic limbs,^[^ ^6^, ^7^, ^8^ ^]^ virtual avatars,^[^ ^9^, ^10^, ^11^ ^]^ consumer digital devices,^[^ ^12^, ^13^, ^14^, ^15^ ^]^ and decoded speech.^[^ ^16^, ^17^, ^18^, ^19^ ^]^ A typical BCI system with a digital device effector is shown in **Figure** **1**. This is an example of an implantable system requiring a percutaneous connection, which has been commonly used in research settings to make seminal engineering and scientific advances in recent decades.^[^ ^19^, ^20^ ^]^

Implantable BCI (iBCI) systems have yielded significant clinical and engineering breakthroughs. This includes the first long‐term independent device use at home (in a controlled, clinical trial setting), computer cursor control, high‐dimensional prosthetic limb control, restoration of own muscle control via functional electrical stimulation or spinal cord stimulation, high‐speed communication output rates, and delivery of somatosensory feedback.^[^ ^18^, ^20^, ^21^, ^22^ ^]^ Although non‐implantable BCI paradigms using EEG have demonstrated clinical benefit,^[^ ^23^ ^]^ and important progress has been made in enabling digital control,^[^ ^24^, ^25^ ^]^ extracranial electrical recording is limited by several factors. These include poor spatial discrimination, low‐pass filtering in the frequency domain resulting from the transmission of signals through the skull, and the requirement for skin abrasion during routine setup of extracranial sensors to enable clinically useable recordings.

Building on advances in implantable BCIs, there are numerous academic and commercial efforts to translate these systems at scale and restore functional independence for individuals with motor impairments as part of routine clinical practice.^[^ ^15^, ^26^ ^]^ However, no iBCI technology has yet received regulatory approval for clinical use or been integrated into standard clinical practice. A major challenge in the clinical translation of iBCI devices is the absence of consensus for clinically meaningful outcome measures that can be used when evaluating device efficacy in clinical trials.^[^ ^27^, ^28^ ^]^ In the USA, the Food and Drug Administration has highlighted the absence of an appropriate outcome measure^[^ ^29^ ^]^ and government funding has been awarded to investigate this.^[^ ^30^ ^]^

In this review, we present the largest and most up‐to‐date estimate of iBCI clinical trial participants worldwide, drawing on data from 112 studies and integrating both published and verified unpublished sources. Unlike previous reviews that include early‐stage applications in contexts such as epilepsy,^[^ ^31^ ^]^ the scope of our analysis is deliberately confined to clinical trials involving target populations with motor impairments, which includes loss of limb function and/or speech. By focusing on individuals with ALS, SCI, and stroke, this review directly addresses the cohorts that stand to benefit most from the clinical translation of iBCI technology. We establish a comprehensive registry not only as a new benchmark for assessing the global status of iBCI trials, but also to serve as an essential resource for stakeholders aiming to advance these technologies toward routine clinical use.

To contextualize the field, we first map the evolution of iBCI research trends, chronicling both the technical advances over time and the heterogeneity in approaches that have contributed to the current challenges. We critically examine a key factor limiting clinical translation, namely the heterogeneity of outcome measures currently used to assess iBCI performance. We propose forward‐looking solutions to the selection of appropriate and standardized outcome measures, intending to extend the discussion to a multidisciplinary audience and accelerate the integration of iBCIs into standard clinical practice.

## 2 Experimental Section

This review was conducted in accordance with the preferred reporting items for systematic reviews and meta‐analysis (PRISMA) 2020 guidelines.^[^ ^32^ ^]^ A completed checklist is included in the Materials SA (Supporting Information). This systematic review was prospectively registered on Open Science Framework (OSF) (https://osf.io/deyrj).

### 2.1 Search Strategy

A sensitive search strategy was developed which combined synonyms for BCIs, intracortical, and patient, in consultation with a senior medical librarian. The search was applied to MEDLINE, Embase and CINAHL using Ovid (Wolters Kluwer, Netherlands), and run from inception to 24th December 2024. The search strategy is included in Materials SB (Supporting Information). Further studies were identified through searching reference lists of included records and searching the Google Scholar profiles of key principal investigators in the field.

### 2.2 Study Selection

Included records were screened by two independent reviewers (ED, ES). An initial pilot screen of 50 records was carried out to ensure concordance, following which reviewers were blinded to each other's decisions using Rayyan (Rayyan Systems Inc, Cambridge, MA, USA). Disagreements were resolved by consensus or discussion with a third reviewer (JB). Full‐text screening was carried out by the same reviewers (ED, ES).

### 2.3 Inclusion and Exclusion Criteria

Studies were considered if they demonstrated the use of implantable BCI devices with an intracranial sensing component and effector. For the purpose of this review, an implantable BCI was defined as an intracranial device which records neural activity representing volitional motor intent, such as intended limb movement or speech, and decoded this into an output signal to control an external effector.^[^ ^25^ ^]^ Non‐implantable BCIs were previously discussed in detail and had a limited potential to provide clinically meaningful restoration of motor function.^[^ ^5^, ^33^, ^34^, ^35^ ^]^ Cochlear implants were sensory prostheses which neither record neural activity nor control external devices, and hence were not included in this study.^[^ ^36^, ^37^ ^]^ Studies conducted in which there was transient device implantation with a primary aim to aid peri‐operative planning, for example, for seizure localization, were considered beyond the scope of this review. All full‐text primary research papers in English were considered. Studies reporting exclusively on non‐human subjects were excluded. An overview of the exclusion hierarchy as provided to reviewers is shown in **Table** **1**.

**Table 1:** Exclusion criteria.

	Exclusion criteria
1	No use of implantable BCI devices
2	Not a full text, independent publication
3	Not a primary study
4	Text not available in English
5	Full text not available
6	Non‐human subjects

### 2.4 Data Management, Extraction, and Appraisal

Records were deduplicated using Rayyan.^[^ ^38^ ^]^ Subsequently, records were blinded and screened by title and abstract by two independent reviewers. Full‐text screening and data extraction were performed using a piloted data extraction proforma in MS Excel. Risk of bias was assessed by two reviewers using the Mixed Methods Appraisal Tool checklist. Reference management was done using Zotero.

### 2.5 Data Synthesis

**Figure 2:**
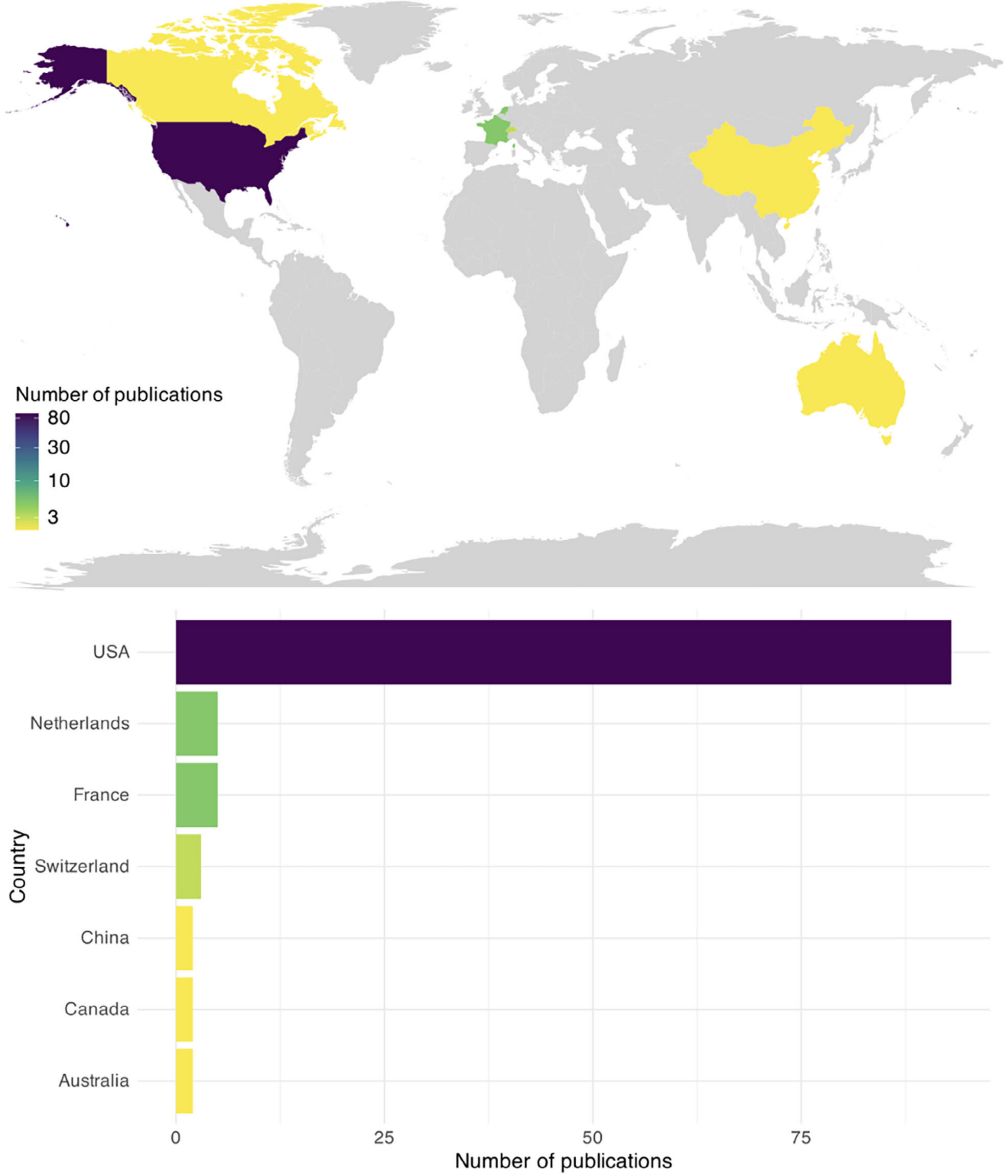
Number of publications by location of research group.

Analysis was performed in R (R Core Team, 2019) using the Tidyverse package. As many participants were reported on in multiple studies, the included studies were cross‐referenced to match the same participants across studies and prevent duplication of demographic details.^[^ ^39^ ^]^ Plots were produced in R using the ggplot2 package.^[^ ^40^ ^]^ Open‐source R code shared in a previous publication was adapted to create the map seen in **Figure** **2**.^[^ ^41^ ^]^ Graphics were produced using Inkscape (Inkscape Project, 2020).

**Figure 3:**
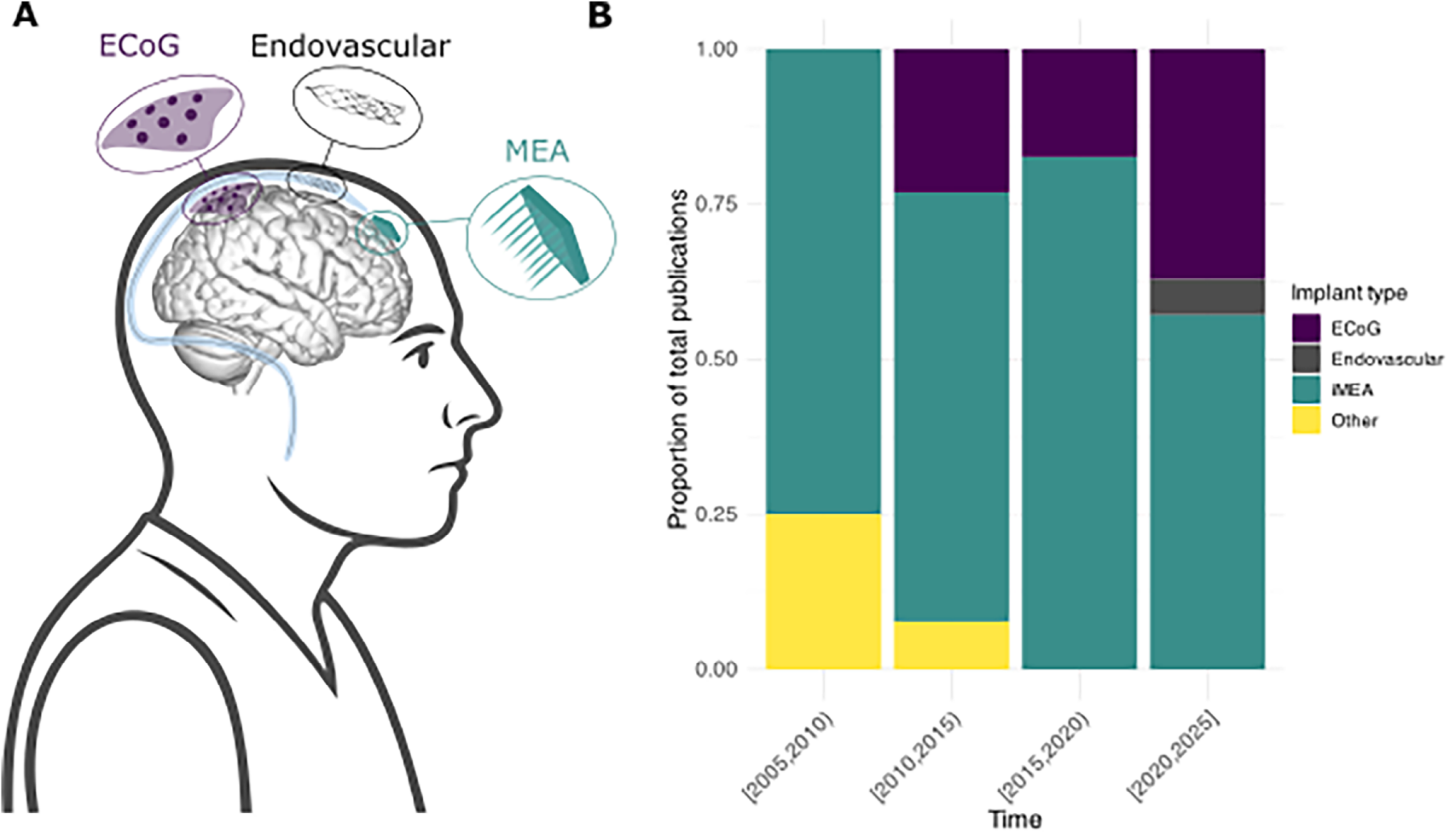
A) Graphic showing ECoG, MEA, and endovascular devices. B) Proportion of total publications using ECoG, endovascular, and MEA‐based sensing over time.

**Figure 4:**
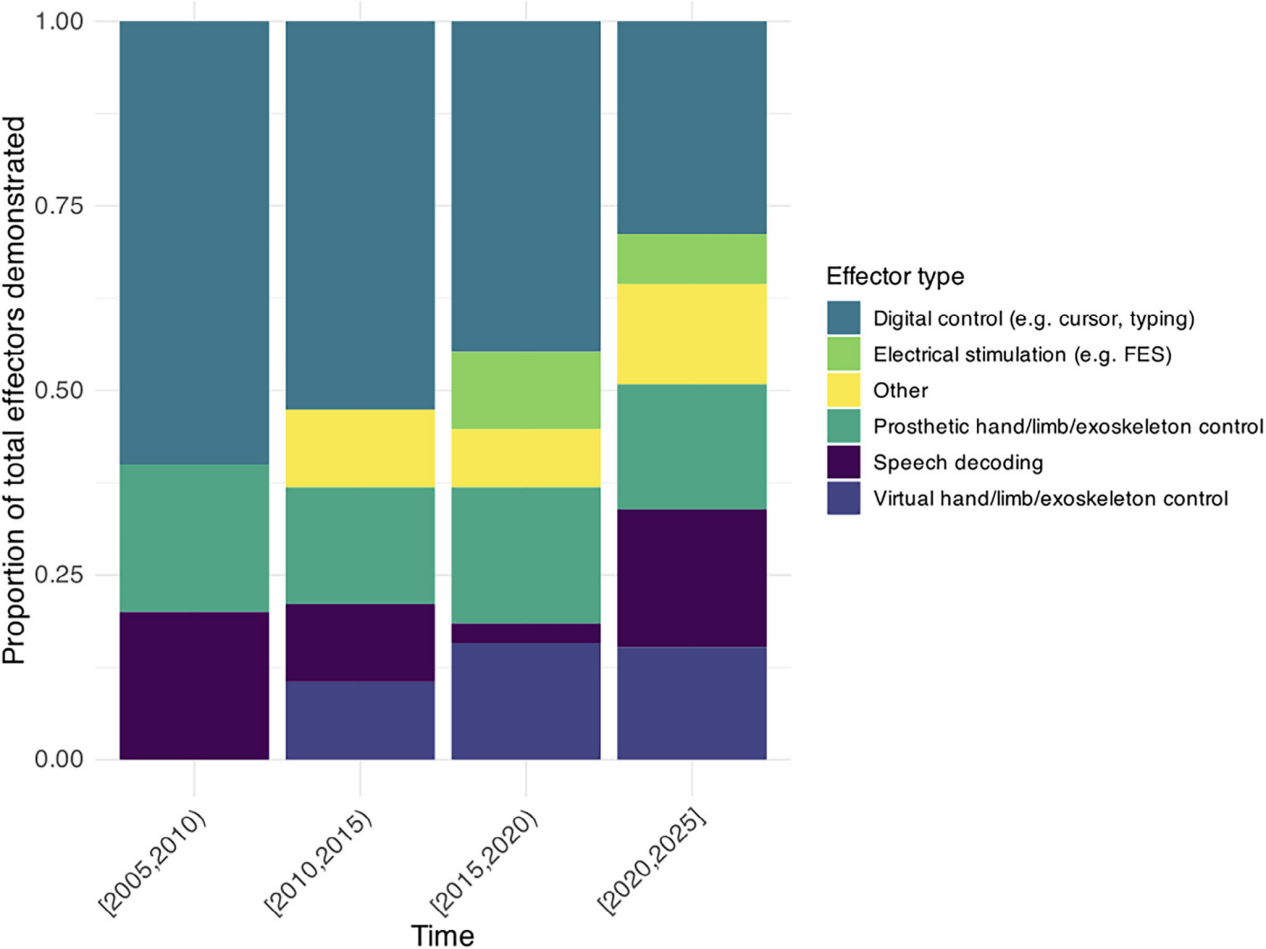
Proportion of publications demonstrating different effectors over time.

Publications were grouped in 5‐year intervals. **Figures** **3** and **4** and 7, which show the proportions of different categories over time, excluded time intervals with ≤3 published studies.

**Figure 5:**
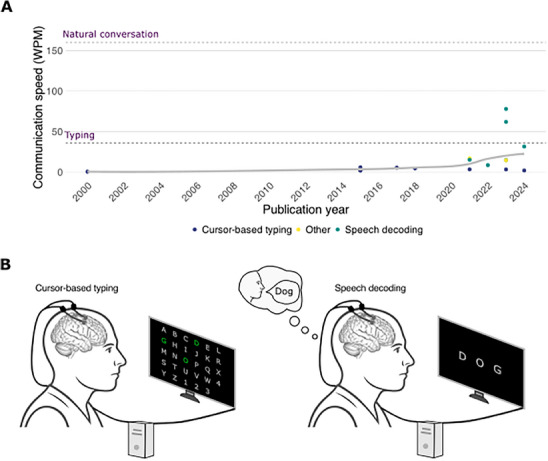
A) Communication speed (WPM) achieved by iBCI publications over time. The speed of human smartphone typing shown for reference. B) A graphic showing two main communication approaches: cursor‐based typing and speech decoding. The graphic in B is loosely adapted from previous graphical representations of speech decoding in refs. [18, 44, 45].

A subgroup analysis was carried out on studies which reported the use of iBCIs for communication purposes, either via cursor‐controlled typing or speech phoneme decoding paradigms. To compare changes in achieved communication speed across studies over time, correct characters per minute (CCPM) estimates were converted to words per minute (WPM) using the commonly used conversion factor of WPM = CPM/5. When speed was reported in terms of characters per minute, it was combined with the reported error rate to calculate an estimate of correct characters per minute. For each participant, the highest achieved mean speed was used, even if aided by additional technology, for example, predictive typing or altered keyboard layouts. Accuracy was averaged across participants. Where error rate was reported, it was converted to accuracy by calculating 100 – error rate. The method with which communication was achieved, that is, cursor‐controlled typing or speech phoneme decoding, is visually indicated in the accompanying figure (**Figure** **5**).

**Figure 6:**
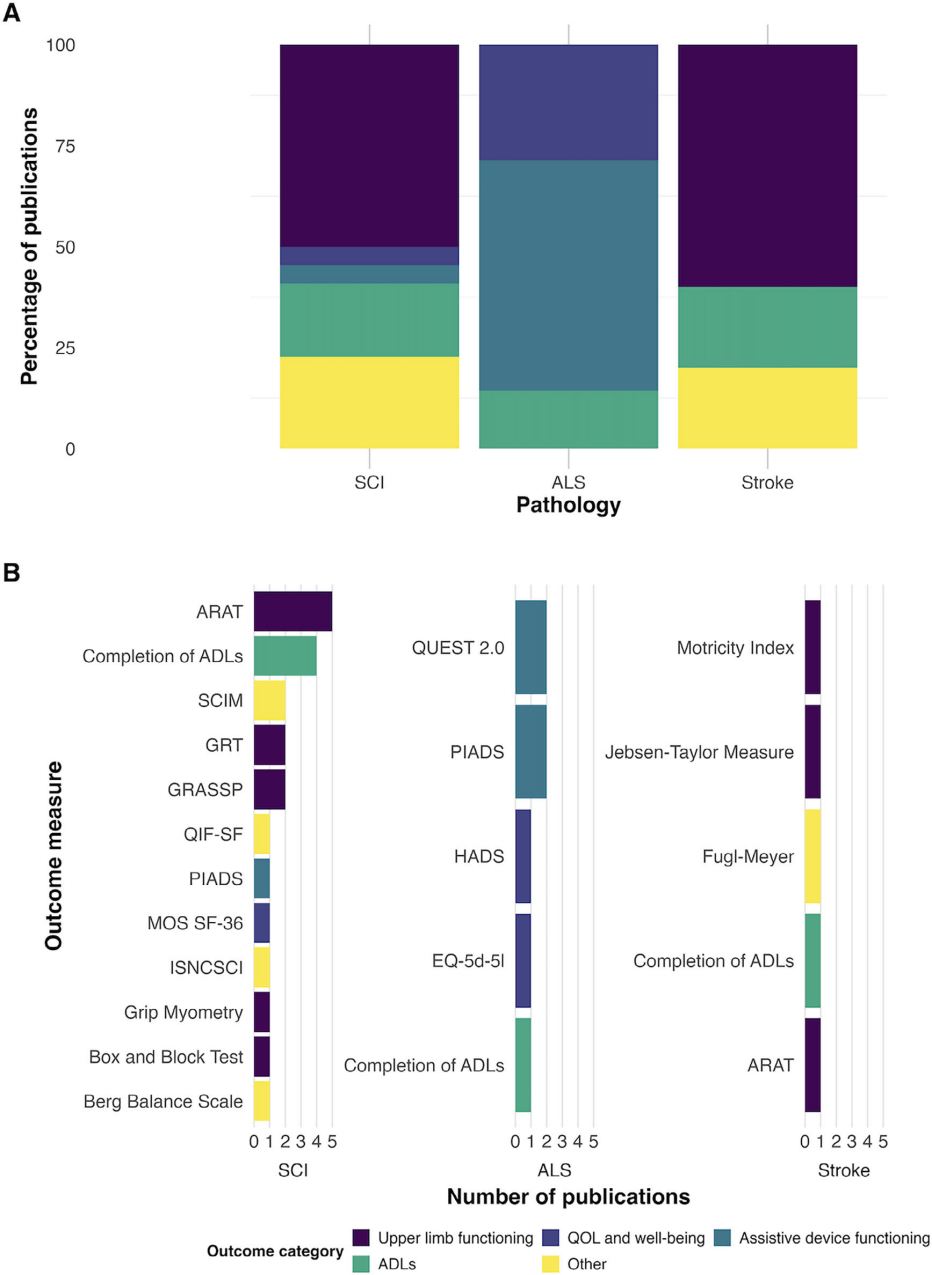
A) Percentage of publications reporting clinical outcome measures, grouped by category, for each target population. B) Number of publications reporting individual clinical outcome measures, by target population. ARAT = Action Research Arm Test. BBS = Berg Balance Scale. BBT = Box and Block Test. EQ‐5d‐5l = EuroQOL‐5d‐5l. FMMIS = Fugl–Meyer Motor Impairment Score. GRASSP = Graded and Redefined Assessment of Strength Sensibility and Prehension. GRT = Grasp Release Test. HADS = Hospital Anxiety and Depression Scale. ISNCSCI = International Standards for Neurological Classification of Spinal Cord Injury. MOS SF‐36 = Medical Outcomes Study Short Form‐36. PIADS = Psychosocial Impact of Assistive Devices Scales. QIF‐SF = Quadriplegia Index of Function – Short Form. QUEST 2.0 = Quebec User Evaluation of Satisfaction with Assistive Technology version 2.0. SCIM = Spinal Cord Independence Measure.

During data extraction, outcome measures were divided into three categories: 1) evaluation of decoder performance (e.g., classifier accuracy), 2) evaluation of task performance (e.g., successful target acquisition), or 3) assessment of a defined clinical outcome (e.g., Graded Redefined Assessment of Strength, Sensibility and Prehension, GRASSP scale). A subgroup analysis was carried out on outcome measures reported for each target population, with the exclusion of adverse event reporting as this was non‐specific to any target population. The results of this analysis are shown in **Figure** **6**.

## 3 Results

### 3.1 Search Results

Our search identified a total of 4639 records across three databases (MEDLINE: 1787; Embase: 2416; CINAHL: 436). Following deduplication, 2966 records remained, of which 382 were selected for full‐text screening. Additionally, 714 records were identified through searches of Google Scholar. A total of 112 studies were included in the final analysis. A full PRISMA flowchart is shown in Materials SC (Supporting Information).

### 3.2 Study Characteristics and Participant Demographics

The review included 112 studies published between 2000–2024. An overview of study characteristics is shown in **Table** **2**. Interest in iBCIs is substantially increasing over time, with nearly half of the publications (49.1%, *n* = 55) published since 2020. Most of the included studies (69.6%, *n* = 78) reported their outcome measures prospectively. Details of all included studies are shown in Materials SD (Supporting Information).

**Table 2:** Characteristics of included studies.

Total	112
Publication year (range)	2000–2024
Study design	
Single‐participant	71 (63.4%)
Multi‐participant	41 (36.6%)
Country	
USA	93
Netherlands	5
France	5
Switzerland	3
China	2
Australia	2
Canada	2
Research group	
BrainGate	43
Brown University	21
Stanford University	12
Case Western Reserve University	8
Massachusetts General Hospital	1
University of California Davis	1
University of Pittsburgh	14
California Institute of Technology	7
Johns Hopkins University	6
Neural Signals Inc., Georgia	6
UMC Utrecht Brain Centre	5
Université Grenoble	5
University of California, San Francisco	4
Battelle Memorial Institute	4
University of Miami	3
Ohio State University	3
University of Lausanne	2
Synchron (University of Melbourne)	2
University of Toronto	2
Zheijiang University	2
Northwestern University, Chicago	1
Thomas Jefferson University, Philadelphia	1
Case Western Reserve University	1
Wyss Center for Bio and Neuroengineering	1

As some research participants were included in multiple publications, we cross‐referenced all 112 studies to identify a total of 57 unique participants with iBCIs in the published literature. A further 23 participants were identified in unpublished literature, including conference presentations, press releases, email communication with PIs of ongoing clinical trials, and blog posts from companies testing iBCIs in clinical trials, bringing the total number of participants with an iBCI globally to 80 individuals globally at the time of print. Data from participants reported on in unpublished literature were not considered in further analyses. An overview of the number of implanted participants identified for each research group is shown in Materials SE (Supporting Information).

The majority (78.9%, *n* = 45) of participants were male, with an average age of 45.4 years. The primary conditions causing motor impairment in these participants were spinal cord injury (SCI, 40.4%, 23 patients), amyotrophic lateral sclerosis (ALS, 31.6%, 18 patients), and stroke (14%, 8 patients). More rare conditions, grouped under “Other” for the purposes of clarity, include spinocerebellar degeneration, mitochondrial myopathy, brachial plexus injury, essential tremor, and chronic pain. An overview of participant demographics is shown in **Table** **3**.

**Table 3:** Demographic data of study participants in the published literature.

Total participants (published literature)	57
Gender	
Male	45 (78.9%)
Female	12 (21.1%)
Age (earliest reported, mean ± SD)	45.4 ± 14.6
Pathology	
Spinal cord injury (SCI)	23 (40.4%)
Amyotrophic lateral sclerosis (ALS)	18 (31.6%)
Stroke	8 (14%)
Other	8 (14%)
Implant type	
Microelectrode array (MEA)	31 (54.4%)
Electrocorticography (ECoG)	16 (28.1%)
Endovascular	4 (7%)
Neurotrophic electrode	6 (10.5%)

### 3.3 Location of Research

Most publications (83%, *n* = 93) were from research groups in the United States, with the BrainGate consortium as the single greatest contributor. Outside of the US, contributions were made from France, the Netherlands, Switzerland, Canada, Australia, and China (Figure 2). We note that 100% of publications published prior to 2010 were from the US, whereas 76.4% of publications after 2020 were from the US, indicating a slight decentralization and geographic diversification, although US dominance remains strong.

### 3.4 Sensor Trends: MEA versus ECoG

The majority of iBCI studies (69.9%, *n* = 78) recorded neural signals using a micro‐electrode array (MEA), commonly the 96‐electrode, silicon‐based Neuroport Electrode (commonly referred to as the “Utah array”, Blackrock Neurotech). MEAs can detect the high‐frequency spikes of single‐unit and multi‐unit activity, representing individual neuronal depolarizations, as well as the slower frequency local field potential (LFP) of the extracellular space surrounding their contacts. ECoG electrodes, which are typically placed subdurally or epidurally, are unable to capture the signals of individual neuronal depolarization events but record oscillatory LFP activity. Historically, ECoG arrays have played a key role in the localization of epileptogenic zones and the delineation of tumor margins, a practice pioneered by Wilder Penfield, fostering the development of a diverse range of commercially available systems today. Most recently, endovascular stent‐based arrays which record ECoG activity have been successfully deployed in human studies.^[^ ^15^, ^42^ ^]^ Of studies published since 2020, 55% of publications report using MEA (*n* = 22) versus 40% ECoG (*n* = 16) and 5% endovascular devices (*n* = 2). The proportion of publications using each implant type over time is visualized in Figure 3.

### 3.5 Effector Trends

BCIs have been trialed using various effectors, each with the ultimate aim of restoring functional independence. Such effectors may be consumer digital devices (e.g., control of a cursor on a computer, tablet, or phone screen), movement of a robotic prosthetic hand or limb, electrical stimulation (e.g., functional electrical stimulation, FES), decoded speech, or movement of a virtual limb or avatar. The earliest publications used primarily digital device effectors, often in the form of cursor control or a center‐out target task on a computer screen. However, with advances in neural decoding and robotics, robotic prosthetic limbs and exoskeletons have been tested. Additionally, virtual effectors (e.g., movement of a virtual avatar) have been demonstrated in recent publications. Of publications since 2020, 28.8% of effectors used in iBCI publications involved digital control, compared to 18.6% speech decoding, 16.9% prosthetic limb/exoskeleton control, 15.3% virtual limb/avatar control, 6.8% electrical stimulation (e.g., FES), and 13.6% other effectors. The changes in effector type in publications over time are visualized in Figure 4.

### 3.6 Communication Speed

Several publications reported attempts to restore communication abilities using an iBCI. Prior to 2020, this was achieved via control of cursors or click‐based spellers to make selections on a visually displayed keyboard. In the last 4 years, several studies have adopted a speech decoding‐based approach instead, using recordings from the areas of the motor cortex corresponding to orofacial muscles and decoding from a pre‐defined vocabulary. Although these are conceptually very different approaches, we were able to compare them using equivalent units. Studies included in this subgroup analysis, including the reported and converted speech, are listed in the Supporting Information.

Using speech decoding, one publication achieved a record communication speed of 78 WPM^[^ ^18^ ^]^ using a 1024‐word vocabulary, surpassing the speed of human smartphone typing of 36 WPM^[^ ^43^ ^]^ and beginning to approach the speed of natural conversation of 160 WPM (45). Another study, published in the same issue of Nature, achieved a slightly lower communication speed of 62 WPM, but with a considerably larger vocabulary of 125 000 words, demonstrating successful large‐vocabulary decoding.^[^ ^44^ ^]^

The achieved communication speed by different publications is shown in Materials SF (Supporting Information).

### 3.7 Types of Outcome Measures Reported

Among the different categories of outcome measures, decoding‐related outcomes such as decoding accuracy were the most frequently reported, in 69.6% of the publications (78 publications). Task‐related outcomes, such as successful task completion or target accuracy, were also reported in most publications (62.5%, *n* = 70). Clinical outcomes were more rarely used, with only 17.9% (20 publications) reporting a clinical outcome.

### 3.8 Clinical Outcome Measures

The clinical outcome measures used varied widely, with 20 different clinical outcomes reported across 20 publications. Exactly half (*n* = 10) of the studies reporting a clinical outcome were published after 2020.

In publications investigating iBCIs in a SCI patient population, clinical outcome measures most commonly focused on upper limb functioning, with measures such as the action research arm test (ARAT) frequently used. By contrast, in publications involving an ALS patient population, no single, specific category of outcome measure predominated, likely because the range of impairments in ALS extends beyond motor function. In these studies, more general outcome measures were used, including evaluations of ADLs, assistive device functioning, and quality of life. This is shown in Figure 6.

### 3.9 Decoding‐ and Task‐Related Outcome Measures

The most commonly reported engineering outcome measure was accuracy, with 74.1% of the included studies (83 publications) reporting this as an outcome. Model accuracy was most commonly reported, for example, decoding or classification accuracy (45.5%, *n* = 51), followed by task accuracy, for example, task success rate or accuracy (42.9%, *n* = 48).

Several studies involved iBCIs developed for the purpose of assisting communication, for example, via cursor‐based typing or speech/phoneme decoding. Out of 112 total studies, 18 studies reported a communication‐speed outcome measure such as correct characters per minute (CCPM) or words per minute (WPM). Of these studies, the majority (11 publications) used character‐based metrics such as CCPM or CPM, although more recent studies (7 publications) tend to use word‐based metrics such as WPM. Another metric of speed, the information transfer rate (ITR) and its derivatives, was reported in 9 studies (8% of all publications).

### 3.10 Funding

**Figure 7:**
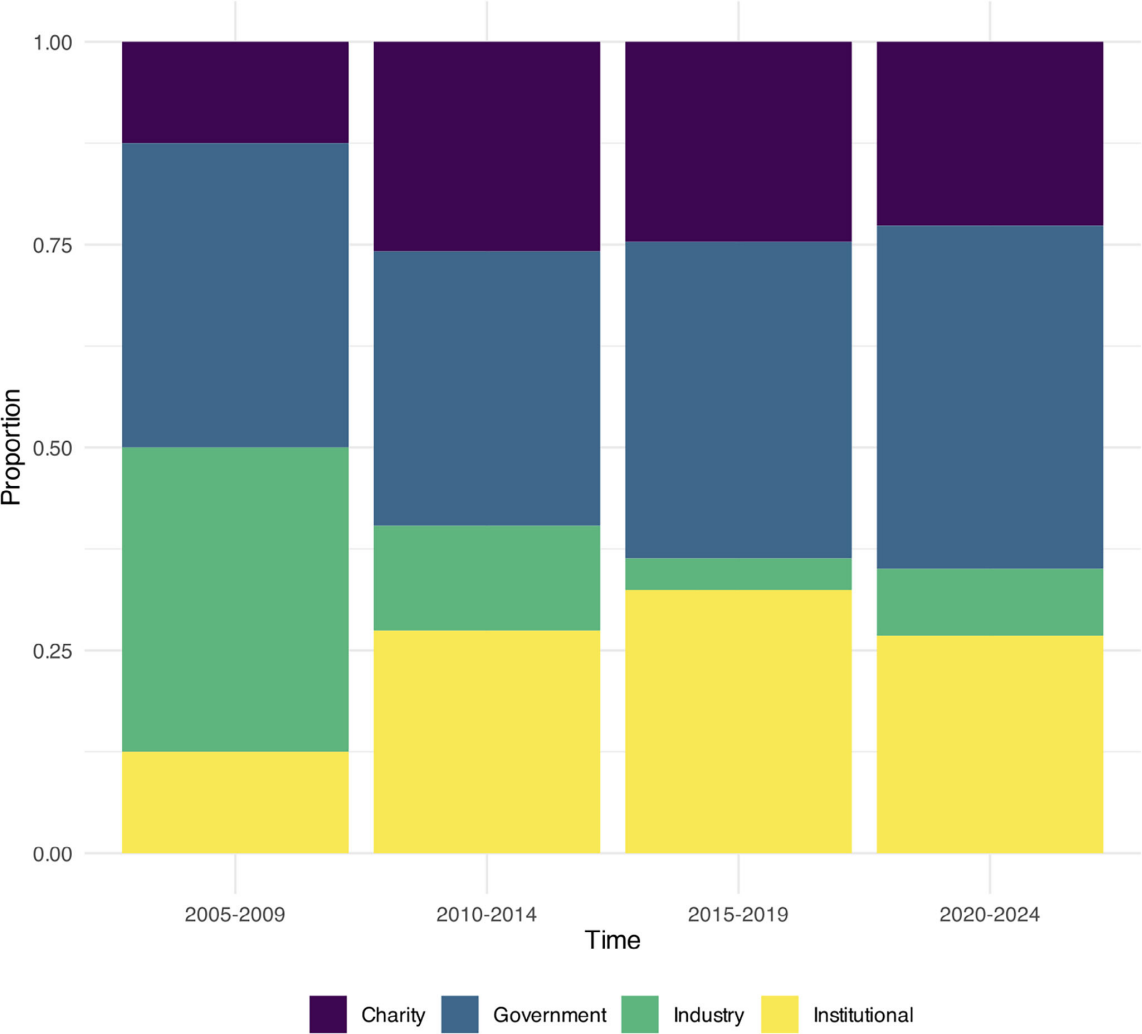
Proportion of funders from different sources over time.

Funding sources were broadly categorized into government, industry, local institutions (e.g., universities), and charity. The quantity of funding was not recorded. As shown in **Figure** **7**, the proportion of studies funded by government sources appears relatively consistent throughout. Out of all funding sources, the proportion of studies funded by industry shows a relative decline in recent years, from 37.5% of publications in 2005–2009 to 8.2% in 2020–2024.

## 4 Discussion

This systematic review characterizes the evolution of motor iBCI research and evaluates outcome measures used to assess device performance. We provide the most up‐to‐date estimate of participants implanted with a BCI, with 80 individuals identified across published and unpublished literature. We found a rapidly increasing research interest in iBCIs, accompanied by an evolving geographic, clinical, and engineering focus. However, few studies used validated clinical outcome measures, and when used, these were heterogeneous and often specific to the types of tasks being performed.

### 4.1 Geographic Trends

Our review demonstrates a substantial majority of iBCI research has been conducted by groups based in the United States. This contrasts with the findings of a recent analysis of primarily non‐implantable BCI studies, which demonstrated a significant and growing proportion of non‐implantable BCI research is performed in China.^[^ ^46^ ^]^ This reflects an advanced iBCI ecosystem cultivated in the USA over decades, benefitting from early government funding^[^ ^47^ ^]^ and the FDA Investigational Device Exemption framework^[^ ^48^ ^]^ and involving long‐term collaboration between clinicians, engineers, scientists, and multiple government agencies.^[^ ^49^ ^]^

Our analysis also revealed some high‐impact output driven by highly localized clusters in Europe and Australia, including groups in Utrecht (the Netherlands), Grenoble (France), and Melbourne (Australia). Such clusters may be enabled by a centralization of engineering, neuroscience, and clinical expertise, which is necessary given the highly multidisciplinary nature of iBCI research.

### 4.2 Evolving Clinical Focus

Our review shows that iBCI devices are primarily researched in individuals with motor impairments due to ALS, spinal cord injury, and stroke. In recent years, we have observed a greater proportion of studies involving patients with more severe clinical impairments affecting both their ability to move limbs and produce speech (a full list of included publications can be found in Materials SD, Supporting Information). Notably, in 2016, Vansteensel and colleagues published the first study using a fully implantable BCI system in an individual with advanced ALS, demonstrating meaningful restoration of function using simple, dependable digital motor outputs.^[^ ^12^ ^]^ This work highlighted the potential for independent home use of BCI devices, particularly in individuals with more severe motor impairments where the need for iBCI technology is clearest. Subsequent publications have continued to build on these findings and involved individuals with more advanced disease.^[^ ^15^, ^16^, ^50^, ^51^ ^]^ Recruitment of individuals with more severe impairments was further influenced by the development of speech iBCIs, which have been studied in individuals with complete dysarthria.^[^ ^18^, ^19^ ^]^ In contrast, earlier iBCI studies mostly involved individuals with SCI and a preserved ability to speak, representing the largest population of individuals with quadriplegic limb impairments.^[^ ^52^ ^]^

With a growing digital ecosystem, there is an increased reliance on digital interfaces to perform ADL including communication, financial management, shopping, leisure, and interacting with healthcare providers. This makes digital access more crucial than ever. Whilst early research primarily focused on restoring physical function in patients with SCI, often with significant funding from the US Department of Veterans Affairs, there has been a recent emphasis on completion of ADL which may be conducted digitally.^[^ ^12^, ^15^, ^50^, ^51^, ^53^ ^]^

Speech iBCIs have made substantial advancements in communication speed by detecting and classifying phonemes and subsequently words using language models during attempted or imagined speech. This reflects the novel development of phoneme classification models, alongside the dramatic increase in computer processing speeds and the incorporation of natural language processing in speech decoder design. In all these publications, decoders were trained and tested using English‐language vocabularies and phoneme sets. It remains uncertain whether decoding performance will translate to tonal languages such as Mandarin, which present classification challenges due to their reliance on pitch and tone to convey meaning.^[^ ^54^ ^]^ Click‐heavy languages such as Xhosa and Zulu may present further challenges for current decoders. Given that a large proportion of the global population speaks tonal languages, future studies must address this issue. To this end, one recent preprint has demonstrated successful decoding of paralinguistic features from intracortical recordings, suggesting the necessary components of language can be decoded from existing recording locations.^[^ ^55^ ^]^

We identified a considerable sex imbalance in the BCI participant population, with over three‐quarters of participants being male. This exceeds the average bias observed in high‐impact clinical research according to recent analyses.^[^ ^56^ ^]^ One likely explanation is the higher incidence of SCI in men, which is nearly twice that in women.^[^ ^57^ ^]^ A similar sex imbalance is seen in ALS, which is 20% more common in men,^[^ ^58^ ^]^ whilst the incidence of stroke is roughly equal between sexes.^[^ ^59^ ^]^ However, female enrollment will be essential to identify or exclude the possibility of any sex‐related differences in iBCI implantation and functioning.

### 4.3 Engineering Trends and Future Potential

One trend highlighted by this review is the increased use and performance of ECoG‐based systems. Despite ECoG signal resolution being limited to local neuron populations rather than individual spikes, these iBCI systems have gained prominence alongside MEA approaches in recent years. Whilst MEA systems can directly record spiking activity from individual neurons, they require cortical penetration, typically sample from smaller cortical areas, and no published study has yet demonstrated the use of a fully implanted MEA iBCI. Historically, the superior spatial resolution of MEA systems has favored their use despite greater clinical invasiveness. However, advances in microfabrication, permitting higher channel counts and improved signal conduction^[^ ^60^, ^61^, ^62^ ^]^ have permitted significant improvements in recording quality and consequent iBCI performance. In 2023, ECoG‐based iBCIs surpassed MEA‐based iBCIs in communication output speed for the first time, although it is notable that the MEA‐based BCI study demonstrated decoding from a significantly larger vocabulary, complicating a direct comparison.^[^ ^18^, ^44^ ^]^ This emergence of studies utilizing ECoG devices has been accompanied by increased reliance on spectral features in neural decoding. Additionally, novel methods of recording have been developed, including endovascular recording with stent‐embedded electrodes, which do not require neurosurgical delivery and may enable access to targets in deep brain regions.^[^ ^15^, ^50^ ^]^

iBCI communication output speeds, often presented as a benchmark of engineering performance, in 2023 surpassed normal typing speeds after years of performance at a substantially lower baseline. Rapid improvements in the performance of communication decoding have been catalyzed by novel decoding paradigms and breakthroughs in understanding the cortical representations of language and speech.^[^ ^19^, ^63^, ^64^ ^]^ Before 2021, the benchmark studies of BCI communication outputs relied on point‐and‐click control of keyboard interfaces to restore typing ability.^[^ ^65^ ^]^ A threefold increase in output speed was first reported as the paradigm shifted to decoding attempted handwriting, transforming the approach from regression control of a computer cursor to a 31‐class classification problem.^[^ ^64^ ^]^ This progress was then far surpassed by decoding attempted speech, focusing on the decoding of attempted phonemes from the area of the motor cortex corresponding to the vocal tract.^[^ ^18^, ^19^, ^44^ ^]^ More than a decade of seminal investigations to characterize the neural basis of speech preceded this work.^[^ ^66^, ^67^ ^]^ We anticipate that the increasing speed of communication using this approach will plateau at the speed of natural conversation (160 words per minute).^[^ ^68^ ^]^ Any future advancements beyond this level will likely require a new paradigm, such as the decoding of semantic information in association cortices.^[^ ^69^ ^]^ However, it has been proposed that the information throughput of human cognition approximates the upper bound of speech output, suggesting the plateau of speech may persist in the long term.^[^ ^70^ ^]^ Even if the speed of communication were limited by this natural threshold, there are still meaningful advancements to be made in other areas to more accurately capture the complexity of human speech, including paralinguistic features such as pitch, cadence, and volume.^[^ ^55^ ^]^ Indeed, iBCI systems must represent all the features of natural human speech in order to fully restore the ability to verbally express oneself.

In addition to these breakthroughs in communication speed, significant progress has been made in developing implantable BCIs to restore locomotion. In a seminal study, Lorach et al. demonstrated reliable recording of locomotor‐related neural signals from an ECoG array over the sensorimotor cortex.^[^ ^71^ ^]^ These signals were decoded and used to trigger epidural electrical stimulation of the spinal cord in a patient with an incomplete cervical SCI. This “brain–spine interface” enabled volitional control of standing and walking, and contributed to neurorehabilitation, with functional improvements persisting even in the absence of stimulation.^[^ ^71^ ^]^

Over the next decade, iBCI systems are expected to advance further, with devices becoming fully implantable with wireless neural telemetry, alongside advanced decoding paradigms that may both increase capabilities and reduce recalibration requirements. Decoder design is continually being improved and enabling improved performance on larger datasets, with an excellent overview of different decoders described in a recent review by Lim et al.^[^ ^31^ ^]^ Novel devices are being developed by industry labs with substantial funding, including Neuralink Corp., Synchron Inc., Paradromics Inc., Precision Neuroscience, Echo Neurotechnologies, and Science Corporation. Both Neuralink and Paradromics are employing an approach using fully implanted intracortical microelectrodes. In a recent blog, Neuralink reported achieving the first fully implantable iMEA‐based system allowing independent at‐home use in 2 individuals.^[^ ^53^ ^]^ Precision Neuroscience is currently investigating its less invasive thin film MEA‐based iBCI, called the Layer 7 Cortical Interface, in two clinical trials, and has recently successfully completed a Series C funding round of 102 million USD.^[^ ^72^, ^73^ ^]^ Synchron has adopted an alternative hardware approach, using a stent electrode device placed endovascularly in the superior sagittal sinus via a minimally invasive neurointerventional procedure. Having received the first investigational device exemption (IDE) from the FDA in 2021, Synchron has implanted its device in 10 patients to date, including 6 patients in the US COMMAND study (NCT05035823).

Despite these advancements, it is notable that the adoption of advanced machine learning approaches to improve decoding has lagged behind other fields.^[^ ^74^, ^75^ ^]^ One key limitation is the relatively small datasets available due to a limited number of human implants and recording sessions. As a consequence, iBCI systems tend to be developed and calibrated on a subject‐by‐subject basis, rather than attempting to train generalized cross‐subject models which utilize advanced neural network architectures. However, non‐invasive data collection methods such as EEG or MEG have facilitated the collection of large datasets in healthy volunteers. One recent study of MEG data applied advanced self‐supervised computer vision methods to MEG data to decode visual perception across individuals, suggesting the feasibility of cross‐subject models for large datasets recorded from iBCI devices.^[^ ^76^ ^]^ As a further challenge, iBCIs require real‐time decoding for optimal functioning, meaning models must be fast, and if run on implanted hardware, they must also be energy efficient. This provides further challenges to the implementation of more computationally intensive algorithms. Finally, there are concerns regarding the explainability of neural network outputs, which are heightened when technologies are deployed clinically.

### 4.4 Industry‐Funded Research Output

An analysis of all funders of iBCI publications reveals a relative decline in the proportion of industry funders over the years. This contrasts with the unprecedented levels of private funding received by commercial iBCI efforts in recent years,^[^ ^77^, ^78^, ^79^ ^]^ relative to government funding.^[^ ^80^, ^81^ ^]^ Possible explanations for a relative decline in industry output are a core focus on a limited number of iBCI applications for scalable translation, the recruitment of larger cohorts as part of single feasibility studies, and the associated increased regulatory burden (NCT05035823). Several companies have chosen to disseminate results via popular media, such as a recent live stream from Neuralink on the social media platform X demonstrating iBCI functioning in their study participant, deviating from the traditional model of scientific publishing.^[^ ^82^ ^]^

In contrast, government funding has remained relatively stable over time, with a consistent proportion of publications reporting government funding. This funding has most often been provided by the United States Government, with substantial contributions from the BRAIN Initiative,^[^ ^83^ ^]^ the Department of Veterans Affairs, and the Defense Advanced Research Projects Agency.^[^ ^47^ ^]^ US Government funding may reduce in the coming years with the substantial reduction of BRAIN initiative funding; however, new funding government sources may also arise, such as the UK Advanced Research and Invention Agency (ARIA) and the US Advanced Research Projects Agency for Health (ARPA‐H).^[^ ^84^, ^85^ ^]^

Although overall funding for iBCI research has risen steadily over the past two decades, the resulting scientific advances have varied depending upon where the funds are directed. DARPA programmes (e.g., *Human Assisted Neural Devices* and *Revolutionizing Prosthetics*) catalyzed foundational breakthroughs in robotic limb control via iBCIs. Subsequent BRAIN Initiative support not only propelled further robotic‐control innovations but also supported fundamental studies of speech production that directly enabled today's speech neuroprostheses. More recently, industry R&D has focused on integrating established decoding capabilities with consumer platforms, translating academic proofs‐of‐concept into devices designed to maximize clinical utility at scale. Nevertheless, despite unprecedented levels of funding in iBCI companies, academic grants remain indispensable for de‐risking novel device concepts and experimental paradigms that cannot attract private funding.

### 4.5 Engineering‐Related Outcome Measures are Consistently Selected

Engineering measures of decoder and task performance were most commonly and consistently used in the included studies. Accuracy was measured in 74.1% of studies (*n* = 83). Performance measures related to speed, for example, characters per minute or bit rate, were utilized in only 16.1% of studies (*n* = 18). The preference for measuring device accuracy as an engineering metric is aligned with patient preference literature, which demonstrates patients prioritize high accuracy over other aspects of iBCI performance, such as speed.^[^ ^86^ ^]^ Whilst commonly used and preferred by patients as a performance characteristic, accuracy is a 1D measure and does not give information on the task difficulty or complexity, for example, the degrees of freedom, cognitive load being used, or training burden. Moreover, isolated measurement of accuracy does not account for the environmental context in which a task can be performed. Therefore, such engineering measures do not account for how an individual feels and functions in their daily life,^[^ ^87^ ^]^ and the US Food and Drug Administration has referred to these assessments as “lab‐tests”, rather than assessments of real‐world function.^[^ ^28^ ^]^ Inclusion of engineering metrics, such as accuracy, may still be useful to evaluate the performance of the BCI device; however, additional assessments of real‐world function will be necessary to determine clinical benefits.

Accuracy was commonly defined at the task level, that is, the number of successful trials/total number of trials, with assessment in 70 studies (62.5%). This definition at the task level is most appropriate for application across different iBCI studies, as it is a standardized accuracy measure for discrete, continuous, and hybrid discrete/continuous iBCI applications. Other definitions, such as classification accuracy, are specific to the nature of the paradigm employed when decoding motor intentions.

Whilst information transfer rate or characters per minute of language output have also been referenced as measures of BCI function,^[^ ^28^ ^]^ these assessments were overrepresented by benchmark studies of iBCI performance, and less commonly used in the literature overall. However, it may be the case that these measures are only reported if successful in demonstrating a new breakthrough, as there is less frequent reporting on low or failed outcome measures across the academic literature.

### 4.6 Clinical iBCI Outcome Measures are Increasingly Utilized, but Highly Heterogenous

Clinical outcome measures were selected more rarely, with only 17.9% (20 studies) reporting a clinical outcome measure. Of these 20 studies, half (50%, *n* = 10) were published since 2020. Despite increasing interest in evaluating the clinical benefit of iBCI devices, there is substantial variability in the assessments being used. This heterogeneity partly reflects differences in target patient populations and the specific functional domains addressed in each study. It is important to note that this is largely driven by the external effectors used in different patient populations, rather than intrinsic differences in patient populations.

For example, studies involving participants with spinal cord injury (SCI) have often focused on attempted control of an upper limb and therefore have employed existing, well‐validated upper extremity functional tests, such as the ARAT, the Graded Redefined Assessment of Strength, Sensibility and Prehension (GRASSP) and the grasp and release test (GRT). This is in line with patient priority research suggesting restoration of arm and hand function is a specific priority for individuals with tetraplegia.^[^ ^88^ ^]^ A closer evaluation of these outcome measures reveals both similarities and key differences. All three are objective performance metrics that quantify aspects of hand and arm function and have been widely applied in study populations with motor impairments, such as those resulting from stroke or spinal cord injury, which are analogous to the target cohorts in iBCI studies. Of these measures, the ARAT provides the most comprehensive evaluation by assessing both fine motor skills (including grasping, gripping, and pinching) and the gross motor movements required for functional tasks. In contrast, the GRASSP extends beyond motor performance to also assess tactile and proprioceptive feedback. This sensory component may make the GRASSP highly suitable for iBCI trials that aim to integrate both motor output and sensory feedback. However, in trials where the intervention is primarily directed at enhancing motor output, the sensory component may add complexity without improving responsiveness. Finally, the GRT is valuable for its simplicity and sensitivity to changes in speed and dexterity during repetitive grasp and release tasks. However, it does not capture broader functional domains such as gross motor strength or (gross motor movements, overall strength) and therefore it is not suitable as a sole outcome measure in iBCI trials aiming to restore movement. Finally, it is worth noting that the Fugl–Meyer assessment, which is frequently used in clinical practice and has been used in an iBCI trial with a stroke patient,^[^ ^89^ ^]^ offers a robust evaluation of motor recovery across both sensory and motor domains following hemiplegic stroke. These existing outcome measures may be useful as part of the “functions” domain of a multi‐component outcome measure framework (see Section 4.7).

Conversely, studies focusing on participants with amyotrophic lateral sclerosis (ALS) tend to use more general assessments, including measures of ADLs, assistive device performance and satisfaction, and quality of life. This is likely a result of their having been fewer efforts to employ prosthetic limbs in ALS iBCI studies, leading to functional assessments centered around ADLs with restored digital device control. Notably, when ADLs were assessed, they were typically evaluated on an activity‐by‐activity basis rather than with a standardized instrument. No ADL measure currently exists that captures restored digital functional independence, despite proposals for a “digital ADL” instrument by Fry et al., and the US Food and Drug Administration. (28,29). Several QoL measures, such as the EuroQol‐5D‐5L, are identified in our review. They benefit from being agnostic to device type and the function being restored. However, QoL assessments are typically used only as supplementary measures when evaluating therapeutic interventions due to inherent limitations. They are often confounded by socioeconomic factors, psychological wellbeing, and comorbidities, and can remain elevated due to the patient's psychological adaptation to deficits.^[^ ^90^ ^]^ Whilst it is necessary to capture patient‐reported outcomes in randomized studies of interventions,^[^ ^91^ ^]^ and a QoL measure may be the most appropriate assessment, these challenges may limit the potential for QoL assessments as a primary measure of clinical benefit with iBCI devices.

### 4.7 Toward a Standardized and Clinically Meaningful iBCI Outcome Measure

Given the inherent heterogeneity in device types, patient selection, and iBCI applications, it is unlikely that one single comprehensive measure will be developed to evaluate overall iBCI clinical benefit. In line with FDA guidance and Fry et al. (2022), BCI clinical outcome measures will likely be structured around three dimensions: how a patient “feels” (e.g., quality of life scores), how a patient “functions” (e.g., ADL scale), and how a patient “survives” (e.g., health‐related outcomes, including device safety).^[^ ^27^ ^]^

In some iBCI applications, existing outcome measures might be used within this framework. For instance, in an iBCI trial aiming to provide a functional benefit by restoring locomotion following spinal cord injury, the 10‐Metre Walk test or Timed Up and Go test could quantify mobility for the “functions” domain. Similarly, iBCI studies focusing on functional upper limb motor restoration in SCI might assess improvements in how a patient “functions” using existing assessments such as the ARAT. Additional outcome measures such as the PIADS or SF‐36 may be used to assess the “feels” domain. How a patient “survives” might be evaluated via systematic monitoring of adverse events, and eventually, in larger cohorts or meta‐analyses, outcomes representing positive, clinically meaningful effects in how a patient survives might be measurable (e.g., cardiovascular benefits). Inclusion of outcome measures assessing caregiver burden, such as the Zarit Burden Interview^[^ ^92^ ^]^ or the Caregiver Reaction Scale^[^ ^93^ ^]^ may also be important both to capture a more holistic impact of iBCIs and can provide valuable insights for reimbursement decisions.

In the near term, however, it is likely that the first wave of clinically translated iBCI devices, for all target populations, will be aimed primarily at restoring control of digital devices and/or communication (e.g., BrainGate2 (NCT00912041), Neuralink PRIME (NCT06429735), Synchron COMMAND (NCT05035823 and BRAVO (NCT03698149). This suggests there is an urgent need for outcome measures capable of capturing improvements in digital functional independence. In addition to a novel digital ADL instrument, another promising development is the concept of a digital motor output, proposed by Sawyer et al. Measuring a digital motor output, that is, the ability of an iBCI to reliably translate a motor intention to a digital output, ensures that any performance measure is agnostic to the external effector device being used in a specific study, and may allow meaningful comparisons between different iBCI systems.^[^ ^94^ ^]^ However, this may not be an appropriate framework to evaluate the performance of speech neuroprostheses, where naturalistic speech can now be directly synthesized from decoding neural signals, without intermediate steps to decode motor kinematics.

The selection and development of appropriate clinical outcome assessments must involve multi‐stakeholder consensus,^[^ ^28^ ^]^ including input from individuals with lived experience of severe motor impairment. This is critical to ensure future device outcomes match a user's priorities and expectations. Moreover, given the emergence of multiple companies working to translate iBCI devices at scale, this work should also ensure collaboration across different commercial actors. In the USA, the creation of the iBCI collaborative community (iBCI‐CC) is an important step to enable this work (ibci‐cc.org), along with the organization of multiple workshops by regulators to discuss iBCI clinical outcome assessments.^[^ ^28^, ^29^ ^]^

### 4.8 The iBCI Registry: an International Registry of iBCI Clinical Trial Participants

In order to encourage collaboration and promote data sharing among research groups and ultimately advance the clinical translation of iBCI technology, we have established an international online registry of iBCI clinical trial participants. This registry contains participant‐level data cross‐referenced from both peer‐reviewed publications and direct communications with research groups, and includes demographic information, device details, and links to publications. The development of this registry follows demand from the field and not only provides the most up‐to‐date estimate of iBCI clinical trial participants globally, but also serves as a dynamic and updatable resource for clinicians, researchers, and regulators. We plan to continuously update and expand this registry, ultimately incorporating open‐access datasets to further facilitate collaborative research, inform regulatory decision‐making, and accelerate the clinical translation of iBCI technologies. The registry will be continually updated and maintained, with a submission form for investigators to share their trial metadata, outcomes, and any associated citations. This registry is freely accessible at *ibciregistry.com*.

### 4.9 Limitations

This review has several limitations that may affect its findings. First, all the studies included were published in English, which may limit an understanding of the global contributions to iBCI research. This is particularly relevant for China, the greatest contributor to natural science research globally, by volume of publications.^[^ ^95^ ^]^ China is also the second most common jurisdiction where BCI patents were filed.^[^ ^96^ ^]^ Much of this research is published in English‐language journals and indexed in major databases such as those searched for this review. However, there is still a possibility that some studies were missed due to language barriers.^[^ ^95^, ^97^ ^]^

The potential for publication bias is another key limitation. The vast majority of published studies are case reports or case series, often focusing on the “best‐performing” patients, which may skew the representation of outcomes. Additionally, commercial interests around iBCI technology may provide an incentive to withhold technological and methodological details.

Finally, the rapidly evolving nature of the iBCI field may limit the longevity of our review's findings, as they may soon become outdated. To address this, we have incorporated discussion of unpublished literature and recent advances, although these were not included in formal analyses. We have also launched the iBCI registry, enabling a dynamically updating estimate of iBCI clinical trial participants worldwide.

## 5 Conclusion

This systematic review highlights the rapid evolution of the implantable BCI field. The past two decades have seen an evolving clinical and technical focus, with most research published in the United States, alongside significant contributions from Europe and China. Sensing technology has advanced considerably, including the increased use of ECoG alongside traditional micro‐electrode arrays. iBCIs have achieved substantial advances in communication speeds, now surpassing the speed of typing and approaching the speed of natural human conversation. Additionally, an expanding range of controllable effectors is enabling the restoration of a broad collection of ADLs. Clinical translation of iBCI devices is now on the horizon. However, to enable regulatory approval, the development and use of novel, clinically relevant outcome measures are vital.

## Conflict of Interest

J.B. reports consulting fees from Synchron Inc. and the UK Advanced Research and Invention Agency (ARIA). E.D. reports consulting fees from Precision Neuroscience.

## Supporting information



Supporting Information

## Data Availability

Data available on reasonable request.
