# Supplementary material for: Toward the Clinical Translation of Implantable Brain–Computer Interfaces for Motor Impairment: Research Trends and Outcome Measures
Source: Adv Sci (Weinh). 2025 Jul 23;12(32):e01912. doi: 10.1002/advs.202501912 (PMC12407270; doi:10.1002/advs.202501912)
Supplement: Supplementary file 1 — Supporting Information [file ADVS-12-e01912-s001.docx]

Supplementary Materials

Towards Clinical Translation of Implantable Brain-Computer Interfaces for Motor Impairment: Research Trends and Outcome Measures

Esmee Dohle MA MB BChir^1^; Eleanor Swanson MSc.^2^; Luka Jovanovic MA MB BChir^3^; Suraya Yusuf MA MB BChir^4^; Lucy Thompson MA BMBCh^5^; Hugo Layard Horsfall BSc MBBS^6,7,8^; William R Muirhead MD BMBS^6,7,8^; Luke Bashford PhD^9,10^; Jamie Brannigan, MA MB BChir^1^

^1^ University of Oxford, Medical Sciences Division, John Radcliffe Hospital, Oxford, UK

^2^ Utrecht University Medical Centre, Utrecht, NL

^3^ East and North Hertfordshire NHS Trust, Lister Hospital, Stevenage, UK

^4^ John Radcliffe Hospital, Headley Way, Oxford, UK

^5^ Faculty of Medicine, Imperial College London, London, UK

^6^ Department of Neurosurgery, National Hospital for Neurology and Neurosurgery, London, UK

^7^ The Francis Crick Institute, London, UK

^8^ Institute of Neurology, University College London, UK

^9^ Department of Neuroscience, Newcastle University, Newcastle upon Tyne, UK

^10^ Department of Neurosurgery, University of Colorado, Denver, USA

**Supplementary materials A: PRISMA checklist**

| **Section and Topic** | **Item #** | **Checklist item** | **Location where item is reported** |
| --- | --- | --- | --- |
| **TITLE** | | |  |
| Title | 1 | Identify the report as a systematic review. | 2 |
| **ABSTRACT** | | |  |
| Abstract | 2 | See the PRISMA 2020 for Abstracts checklist. | 2 |
| **INTRODUCTION** | | |  |
| Rationale | 3 | Describe the rationale for the review in the context of existing knowledge. | 3-5 |
| Objectives | 4 | Provide an explicit statement of the objective(s) or question(s) the review addresses. | 5 |
| **METHODS** | | |  |
| Eligibility criteria | 5 | Specify the inclusion and exclusion criteria for the review and how studies were grouped for the syntheses. | 7 |
| Information sources | 6 | Specify all databases, registers, websites, organisations, reference lists and other sources searched or consulted to identify studies. Specify the date when each source was last searched or consulted. | 6 |
| Search strategy | 7 | Present the full search strategies for all databases, registers and websites, including any filters and limits used. | Supplementary Materials |
| Selection process | 8 | Specify the methods used to decide whether a study met the inclusion criteria of the review, including how many reviewers screened each record and each report retrieved, whether they worked independently, and if applicable, details of automation tools used in the process. | 6-8 |
| Data collection process | 9 | Specify the methods used to collect data from reports, including how many reviewers collected data from each report, whether they worked independently, any processes for obtaining or confirming data from study investigators, and if applicable, details of automation tools used in the process. | 6-8 |
| Data items | 10a | List and define all outcomes for which data were sought. Specify whether all results that were compatible with each outcome domain in each study were sought (e.g. for all measures, time points, analyses), and if not, the methods used to decide which results to collect. | 6-8 |
|  | 10b | List and define all other variables for which data were sought (e.g. participant and intervention characteristics, funding sources). Describe any assumptions made about any missing or unclear information. | 6-8 |
| Study risk of bias assessment | 11 | Specify the methods used to assess risk of bias in the included studies, including details of the tool(s) used, how many reviewers assessed each study and whether they worked independently, and if applicable, details of automation tools used in the process. | 6-8 |
| Effect measures | 12 | Specify for each outcome the effect measure(s) (e.g. risk ratio, mean difference) used in the synthesis or presentation of results. | N/A |
| Synthesis methods | 13a | Describe the processes used to decide which studies were eligible for each synthesis (e.g. tabulating the study intervention characteristics and comparing against the planned groups for each synthesis (item #5)). | 6-8 |
|  | 13b | Describe any methods required to prepare the data for presentation or synthesis, such as handling of missing summary statistics, or data conversions. | 6-8 |
|  | 13c | Describe any methods used to tabulate or visually display results of individual studies and syntheses. | 6-8 |
|  | 13d | Describe any methods used to synthesize results and provide a rationale for the choice(s). If meta-analysis was performed, describe the model(s), method(s) to identify the presence and extent of statistical heterogeneity, and software package(s) used. | 6-8 |
|  | 13e | Describe any methods used to explore possible causes of heterogeneity among study results (e.g. subgroup analysis, meta-regression). | 6-8 |
|  | 13f | Describe any sensitivity analyses conducted to assess robustness of the synthesized results. | N/A |
| Reporting bias assessment | 14 | Describe any methods used to assess risk of bias due to missing results in a synthesis (arising from reporting biases). | 6-8 |
| Certainty assessment | 15 | Describe any methods used to assess certainty (or confidence) in the body of evidence for an outcome. | 6-8 |
| **RESULTS** | | |  |
| Study selection | 16a | Describe the results of the search and selection process, from the number of records identified in the search to the number of studies included in the review, ideally using a flow diagram. | 9, Supplementary Materials |
|  | 16b | Cite studies that might appear to meet the inclusion criteria, but which were excluded, and explain why they were excluded. | 6-7 |
| Study characteristics | 17 | Cite each included study and present its characteristics. | 10-12, Supplementary Materials |
| Risk of bias in studies | 18 | Present assessments of risk of bias for each included study. | Supplementary Materials |
| Results of individual studies | 19 | For all outcomes, present, for each study: (a) summary statistics for each group (where appropriate) and (b) an effect estimate and its precision (e.g. confidence/credible interval), ideally using structured tables or plots. | N/A (No meta-analysis) |
| Results of syntheses | 20a | For each synthesis, briefly summarise the characteristics and risk of bias among contributing studies. | Supplementary Materials (MMAT) |
|  | 20b | Present results of all statistical syntheses conducted. If meta-analysis was done, present for each the summary estimate and its precision (e.g. confidence/credible interval) and measures of statistical heterogeneity. If comparing groups, describe the direction of the effect. | 9-21 |
|  | 20c | Present results of all investigations of possible causes of heterogeneity among study results. | N/A |
|  | 20d | Present results of all sensitivity analyses conducted to assess the robustness of the synthesized results. | N/A |
| Reporting biases | 21 | Present assessments of risk of bias due to missing results (arising from reporting biases) for each synthesis assessed. | Supplementary Materials |
| Certainty of evidence | 22 | Present assessments of certainty (or confidence) in the body of evidence for each outcome assessed. |  |
| **DISCUSSION** | | |  |
| Discussion | 23a | Provide a general interpretation of the results in the context of other evidence. | 22-32 |
|  | 23b | Discuss any limitations of the evidence included in the review. | 31-32 |
|  | 23c | Discuss any limitations of the review processes used. | 31-32 |
|  | 23d | Discuss implications of the results for practice, policy, and future research. | 22-32 |
| **OTHER INFORMATION** | | |  |
| Registration and protocol | 24a | Provide registration information for the review, including register name and registration number, or state that the review was not registered. | 6 |
|  | 24b | Indicate where the review protocol can be accessed, or state that a protocol was not prepared. | 6 |
|  | 24c | Describe and explain any amendments to information provided at registration or in the protocol. | N/A |
| Support | 25 | Describe sources of financial or non-financial support for the review, and the role of the funders or sponsors in the review. | 2 |
| Competing interests | 26 | Declare any competing interests of review authors. | 32 |
| Availability of data, code and other materials | 27 | Report which of the following are publicly available and where they can be found: template data collection forms; data extracted from included studies; data used for all analyses; analytic code; any other materials used in the review. | 32 |

*From:*  Page MJ, McKenzie JE, Bossuyt PM, Boutron I, Hoffmann TC, Mulrow CD, et al. The PRISMA 2020 statement: an updated guideline for reporting systematic reviews. BMJ 2021;372:n71. doi: 10.1136/bmj.n71

**Supplementary materials B: Example search strategy as applied to MEDLINE**

1 Brain-Computer Interfaces/ 4689

2 brain computer interface*.mp. 8312

3 BCI*.mp. 7982

4 brain machine interface*.mp. 1790

5 neural prosthe*.mp. 1346

6 neuroprosthe*.mp. 2075

7 neuromotor prosthe*.mp. 19

8 1 or 2 or 3 or 4 or 5 or 6 or 7 14387

9 implant*.mp. 608877

10 "prostheses and implants"/ 50025

11 electrodes, implanted/ 21495

12 neural prostheses/ 503

13 intracortical.mp. 7143

14 microelectrode array*.mp. 2730

15 MEA.mp. 4324

16 (Utah adj2 array).mp. 72

17 electrocorticography.mp. 3409

18 ecog.mp. 10740

19 stentrode*.mp. 19

20 endovascular.mp. 75471

21 9 or 10 or 11 or 12 or 13 or 14 or 15 or 16 or 17 or 18 or 19 or 20 689516

22 patient*.mp. 8670314

23 user*.mp. 299593

24 individual*.mp. 2031510

25 exp Patients/ 84088

26 22 or 23 or 24 or 25 10195016

27 8 and 21 and 26 1652

**Supplementary materials C: PRISMA flowchart**

**
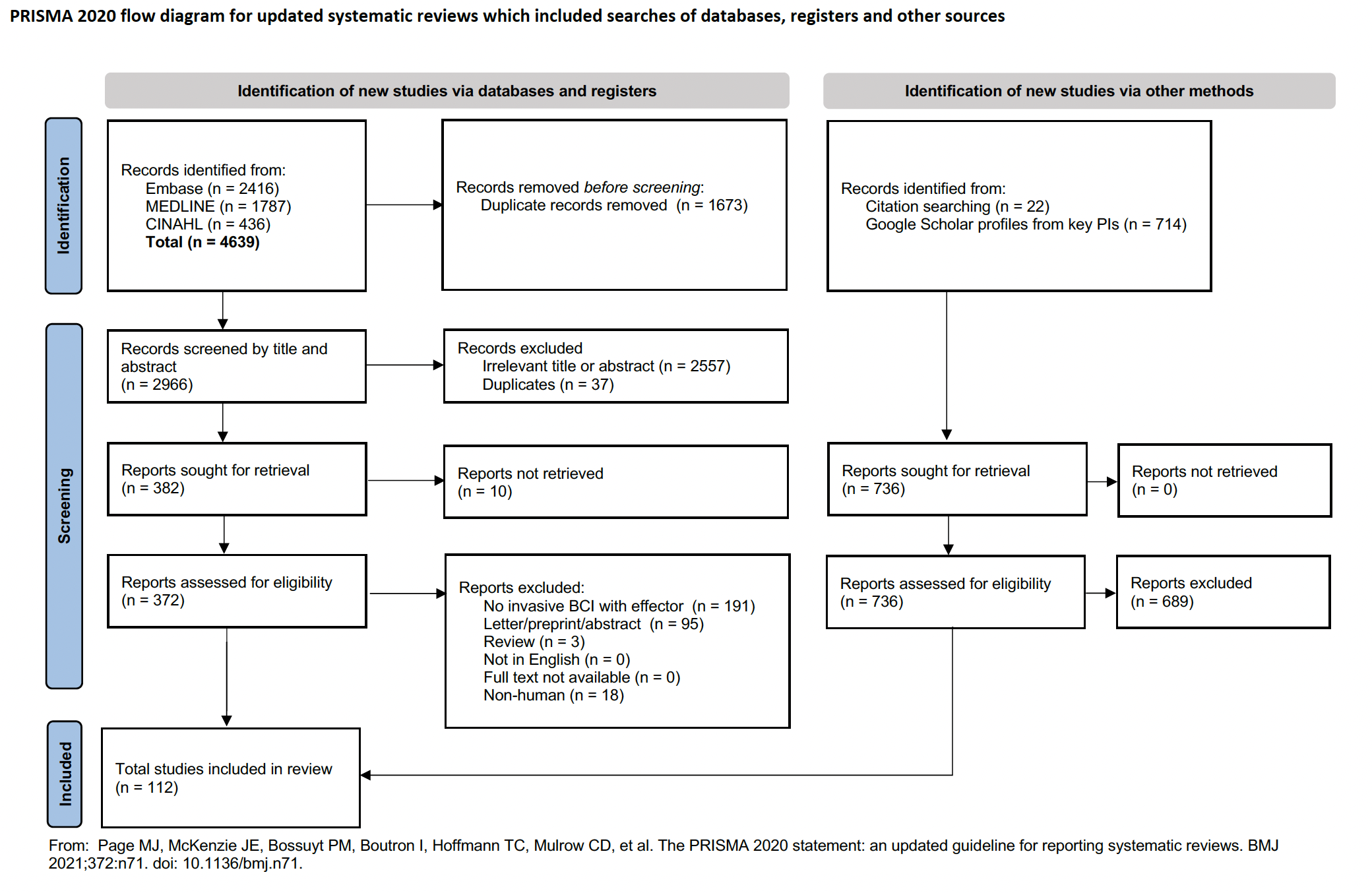
**

**Supplementary materials D: Table of included studies**

| **Year** | **Authors** | **Study title** |
| --- | --- | --- |
| 2015 | (Aflalo et al., 2015) | Neurophysiology. Decoding motor imagery from the posterior parietal cortex of a tetraplegic human. |
| 2017 | (Ajiboye et al., 2017) | Restoration of reaching and grasping movements through brain-controlled muscle stimulation in a person with tetraplegia: a proof-of-concept demonstration. |
| 2012 | (Ajiboye et al., 2012) | Prediction of imagined single-joint movements in a person with high-level tetraplegia |
| 2024 | (Angrick et al., 2024) | Online speech synthesis using a chronically implanted brain-computer interface in an individual with ALS. |
| 2015 | (Bacher et al., 2015) | Neural Point-and-Click Communication by a Person With Incomplete Locked-In Syndrome. |
| 2024 | (Bashford et al., 2024) | Neural subspaces of imagined movements in parietal cortex remain stable over several years in humans. |
| 2019 | (Benabid et al., 2019) | An exoskeleton controlled by an epidural wireless brain-machine interface in a tetraplegic patient: a proof-of-concept demonstration. |
| 2019 | (Bockbrader et al., 2019) | Clinically Significant Gains in Skillful Grasp Coordination by an Individual With Tetraplegia Using an Implanted Brain-Computer Interface With Forearm Transcutaneous Muscle Stimulation. |
| 2016 | (Bouton et al., 2016) | Restoring cortical control of functional movement in a human with quadriplegia |
| 2018 | (Brandman et al., 2018a) | Robust Closed-Loop Control of a Cursor in a Person with Tetraplegia using Gaussian Process Regression. |
| 2018 | (Brandman et al., 2018b) | Rapid calibration of an intracortical brain-computer interface for people with tetraplegia. |
| 2011 | (Brumberg et al., 2011, p. 20) | Classification of intended phoneme production from chronic intracortical microelectrode recordings in speech-motor cortex |
| 2021 | (Cajigas et al., 2021) | Implantable brain–computer interface for neuroprosthetic-enabled volitional hand grasp restoration in spinal cord injury |
| 2023 | (Cajigas et al., 2023) | Brain-Computer interface control of stepping from invasive electrocorticography upper-limb motor imagery in a patient with quadriplegia. |
| 2024 | (Candrea et al., 2024) | A click-based electrocorticographic brain-computer interface enables long-term high-performance switch scan spelling. |
| 2024 | (Card et al., 2024) | An accurate and rapidly calibrating speech neuroprosthesis. |
| 2011 | (Chadwick et al., 2011) | Continuous neuronal ensemble control of simulated arm reaching by a human with tetraplegia |
| 2022 | (Chaudhary et al., 2022) | Spelling interface using intracortical signals in a completely locked-in patient enabled via auditory neurofeedback training |
| 2021 | (Colachis et al., 2021) | Long-term intracortical microelectrode array performance in a human: a 5 year retrospective analysis |
| 2018 | (Colachis et al., 2018) | Dexterous control of seven functional hand movements using cortically-controlled transcutaneous muscle stimulation in a person with tetraplegia |
| 2013 | (Collinger et al., 2013) | High-performance neuroprosthetic control by an individual with tetraplegia. |
| 2022 | (Davis et al., 2022) | Design-development of an at-home modular brain-computer interface (BCI) platform in a case study of cervical spinal cord injury. |
| 2018 | (Degenhart et al., 2018) | Remapping cortical modulation for brain-machine interfaces: A somatotopy-based approach in individuals with upper-limb paralysis |
| 2021 | (Dekleva et al., 2021) | Generalizable cursor click decoding using grasp-related neural transients. |
| 2024 | (Dekleva et al., 2024, p. 20) | Motor cortex retains and reorients neural dynamics during motor imagery |
| 2021 | (Deo et al., 2021) | Effects of peripheral haptic feedback on intracortical brain-computer interface control and associated sensory responses in motor cortex |
| 2016 | (Downey et al., 2016) | Blending of brain-machine interface and vision-guided autonomous robotics improves neuroprosthetic arm performance during grasping |
| 2018 | (Downey et al., 2018) | Implicit Grasp Force Representation in Human Motor Cortical Recordings. |
| 2017 | (Downey et al., 2017) | Motor cortical activity changes during neuroprosthetic-controlled object interaction |
| 2023 | (Fan et al., 2023) | Plug-and-Play Stability for Intracortical Brain-Computer Interfaces: A One-Year Demonstration of Seamless Brain-to-Text Communication. |
| 2022 | (Feng et al., 2022) | Design a Novel BCI for Neurorehabilitation Using Concurrent LFP and EEG Features: A Case Study. |
| 2021 | (Flesher et al., 2021) | A brain-computer interface that evokes tactile sensations improves robotic arm control |
| 2019 | (Freudenburg et al., 2019) | Sensorimotor ECoG Signal Features for BCI Control: A Comparison Between People With Locked-In Syndrome and Able-Bodied Controls |
| 2017 | (Friedenberg et al., 2017) | Neuroprosthetic-enabled control of graded arm muscle contraction in a paralyzed human. |
| 2022 | (Ganesh et al., 2022) | Slow Firing Single Units Are Essential for Optimal Decoding of Silent Speech |
| 2015 | (Gilja et al., 2015) | Clinical translation of a high-performance neural prosthesis. |
| 2023 | (Guan et al., 2023, p. 202) | Decoding and geometry of ten finger movements in human posterior parietal cortex and motor cortex. |
| 2022 | (Guan et al., 2022) | Stability of motor representations after paralysis. |
| 2009 | (Guenther et al., 2009) | A wireless brain-machine interface for real-time speech synthesis. |
| 2022 | (Guthrie et al., 2022) | The impact of distractions on intracortical brain–computer interface control of a robotic arm |
| 2022 | (Handelman et al., 2022) | Shared Control of Bimanual Robotic Limbs With a Brain-Machine Interface for Self-Feeding. |
| 2024 | (Herring et al., 2024) | Reconnecting the Hand and Arm to the Brain: Efficacy of Neural Interfaces for Sensorimotor Restoration After Tetraplegia |
| 2006 | (Hochberg et al., 2006) | Neuronal ensemble control of prosthetic devices by a human with tetraplegia. |
| 2012 | (Hochberg et al., 2012) | Reach and grasp by people with tetraplegia using a neurally controlled robotic arm. |
| 2015 | (Jarosiewicz et al., 2015) | Virtual typing by people with tetraplegia using a self-calibrating intracortical brain-computer interface. |
| 2016 | (Jarosiewicz et al., 2016) | Retrospectively supervised click decoder calibration for self-calibrating point-and-click brain-computer interfaces. |
| 2013 | (Jarosiewicz et al., 2013) | Advantages of closed-loop calibration in intracortical brain-computer interfaces for people with tetraplegia |
| 2022 | (Jiang et al., 2022) | Short report: surgery for implantable brain-computer interface assisted by robotic navigation system. |
| 2020 | (Jorge et al., 2020) | Classification of Individual Finger Movements Using Intracortical Recordings in Human Motor Cortex. |
| 2004 | (Kennedy et al., 2004) | Computer control using human intracortical local field potentials |
| 2000 | (Kennedy et al., 2000) | Direct control of a computer from the human central nervous system. |
| 2011 | (Kennedy et al., 2011) | Making the lifetime connection between brain and machine for restoring and enhancing function. |
| 2011 | (Kim et al., 2011) | Point-and-click cursor control with an intracortical neural interface system by humans with tetraplegia. |
| 2008 | (Kim et al., 2008) | Neural control of computer cursor velocity by decoding motor cortical spiking activity in humans with tetraplegia |
| 2017 | (Kryger et al., 2017) | Flight simulation using a Brain-Computer Interface: A pilot, pilot study. |
| 2021 | (Larzabal et al., 2021) | Long-term stability of the chronic epidural wireless recorder WIMAGINE in tetraplegic patients |
| 2020 | (Leinders et al., 2020) | Dorsolateral prefrontal cortex-based control with an implanted brain-computer interface. |
| 2023 | (Lorach et al., 2023) | Walking naturally after spinal cord injury using a brain-spine interface. |
| 2023 | (Luo et al., 2023) | Stable Decoding from a Speech BCI Enables Control for an Individual with ALS without Recalibration for 3 Months. |
| 2012 | (Márquez-Chin et al., 2012) | Real-time two-dimensional asynchronous control of a computer cursor with a single subdural electrode. |
| 2014 | (Masse et al., 2014) | Non-causal spike filtering improves decoding of movement intention for intracortical BCIs |
| 2022 | (Metzger et al., 2022) | Generalizable spelling using a speech neuroprosthesis in an individual with severe limb and vocal paralysis |
| 2023 | (Metzger et al., 2023) | A high-performance neuroprosthesis for speech decoding and avatar control |
| 2018 | (Milekovic et al., 2018) | Stable long-term BCI-enabled communication in ALS and locked-in syndrome using LFP signals. |
| 2019 | (Milekovic et al., 2019) | Volitional control of single-electrode high gamma local field potentials by people with paralysis |
| 2023 | (Mitchell et al., 2023) | Assessment of Safety of a Fully Implanted Endovascular Brain-Computer Interface for Severe Paralysis in 4 Patients: The Stentrode With Thought-Controlled Digital Switch (SWITCH) Study. |
| 2022 | (Moly et al., 2022) | An adaptive closed-loop ECoG decoder for long-term and stable bimanual control of an exoskeleton by a tetraplegic. |
| 2021 | (Moses et al., 2021) | Neuroprosthesis for Decoding Speech in a Paralyzed Person with Anarthria |
| 2022 | (Nickl et al., 2022) | Characteristics and stability of sensorimotor activity driven by isolated-muscle group activation in a human with tetraplegia |
| 2018 | (Nuyujukian et al., 2018) | Cortical control of a tablet computer by people with paralysis |
| 2021 | (Oxley et al., 2021) | Motor neuroprosthesis implanted with neurointerventional surgery improves capacity for activities of daily living tasks in severe paralysis: first in-human experience |
| 2017 | (Pandarinath et al., 2017) | High performance communication by people with paralysis using an intracortical brain-computer interface |
| 2015 | (Pandarinath et al., 2015) | Neural population dynamics in human motor cortex during movements in people with ALS |
| 2019 | (Pels et al., 2019) | Stability of a chronic implanted brain-computer interface in late-stage amyotrophic lateral sclerosis. |
| 2013 | (Perge et al., 2013) | Intra-day signal instabilities affect decoding performance in an intracortical neural interface system. |
| 2014 | (Perge et al., 2014) | Reliability of directional information in unsorted spikes and local field potentials recorded in human motor cortex. |
| 2020 | (Rastogi et al., 2020) | Neural Representation of Observed, Imagined, and Attempted Grasping Force in Motor Cortex of Individuals with Chronic Tetraplegia |
| 2021 | (Rastogi et al., 2021) | The Neural Representation of Force across Grasp Types in Motor Cortex of Humans with Tetraplegia |
| 2023 | (Rizzoglio et al., 2023) | From monkeys to humans: observation-basedEMGbrain-computer interface decoders for humans with paralysis. |
| 2022 | (Rouanne et al., 2022) | Unsupervised adaptation of an ECoG based brain-computer interface using neural correlates of task performance. |
| 2023 | (Rubin et al., 2023) | Interim Safety Profile From the Feasibility Study of the BrainGate Neural Interface System. |
| 2022 | (Rubin et al., 2022) | Learned Motor Patterns Are Replayed in Human Motor Cortex during Sleep |
| 2019 | (Sakellaridi et al., 2019) | Intrinsic Variable Learning for Brain-Machine Interface Control by Human Anterior Intraparietal Cortex. |
| 2022 | (Serino et al., 2022) | Sense of agency for intracortical brain-machine interfaces. |
| 2022 | (Serruya et al., 2022) | Neuromotor prosthetic to treat stroke-related paresis: N-of-1 trial. |
| 2023 | (Shah et al., 2023) | A brain-computer typing interface using finger movements. |
| 2013 | (Shaikhouni et al., 2013) | Somatosensory responses in a human motor cortex |
| 2016 | (Sharma et al., 2016) | Using an Artificial Neural Bypass to Restore Cortical Control of Rhythmic Movements in a Human with Quadriplegia |
| 2021 | (Silversmith et al., 2021) | Plug-and-play control of a brain–computer interface through neural map stabilization |
| 2011 | (Simeral et al., 2011) | Neural control of cursor trajectory and click by a human with tetraplegia 1000 days after implant of an intracortical microelectrode array. |
| 2021 | (Simeral et al., 2021) | Home Use of a Percutaneous Wireless Intracortical Brain-Computer Interface by Individuals With Tetraplegia. |
| 2022 | (Śliwowski et al., 2022) | Decoding ECoG signal into 3D hand translation using deep learning. |
| 2019 | (Stavisky et al., 2019) | Neural ensemble dynamics in dorsal motor cortex during speech in people with paralysis |
| 2020 | (Stavisky et al., 2020) | Speech-related dorsal motor cortex activity does not interfere with iBCI cursor control. |
| 2017 | (Talakoub et al., 2017) | Reconstruction of upper limb movement from electrocorticographic signals to control functional electrical stimulation for hand function restoration |
| 2008 | (Truccolo et al., 2008) | Primary Motor Cortex Tuning to Intended Movement Kinematics in Humans with Tetraplegia |
| 2016 | (Vansteensel et al., 2016) | Fully Implanted Brain-Computer Interface in a Locked-In Patient with ALS. |
| 2024 | (Vansteensel et al., 2024) | Longevity of a Brain-Computer Interface for Amyotrophic Lateral Sclerosis |
| 2018 | (Vargas-Irwin et al., 2018) | Watch, Imagine, Attempt: Motor Cortex Single-Unit Activity Reveals Context-Dependent Movement Encoding in Humans With Tetraplegia |
| 2022 | (WANDELT et al., 2022) | Decoding grasp and speech signals from the cortical grasp circuit in a tetraplegic human |
| 2013 | (Wang et al., 2013) | An electrocorticographic brain interface in an individual with tetraplegia. |
| 2019 | (Weiss et al., 2019) | Demonstration of a portable intracortical brain-computer interface |
| 2019 | (Willett et al., 2019) | Principled BCI Decoder Design and Parameter Selection Using a Feedback Control Model. |
| 2021 | (Willett et al., 2021) | High-performance brain-to-text communication via handwriting |
| 2023 | (Willett et al., 2023) | A high-performance speech neuroprosthesis |
| 2020 | (Wilson et al., 2020) | Decoding spoken English from intracortical electrode arrays in dorsal precentral gyrus. |
| 2014 | (Wodlinger et al., 2014) | Ten-dimensional anthropomorphic arm control in a human brain-machine interface: difficulties, solutions, and limitations |
| 2024 | (Wyse-Sookoo et al., 2024) | Stability of ECoG high gamma signals during speech and implications for a speech BCI system in an individual with ALS: a year-long longitudinal study. |
| 2018 | (YOUNG et al., 2018) | Signal processing methods for reducing artifacts in microelectrode brain recordings caused by functional electrical stimulation |
| 2019 | (Young et al., 2019) | Closed-loop cortical control of virtual reach and posture using Cartesian and joint velocity commands |
| 2020 | (Zhang et al., 2020) | Preservation of Partially Mixed Selectivity in Human Posterior Parietal Cortex across Changes in Task Context. |
| 2018 | (Zhang et al., 2018) | Extracting wavelet based neural features from human intracortical recordings for neuroprosthetics applications |

**Supplementary Materials E: Number of implanted participants identified by research group/company**

| **Group** | **Total participants** | **Type of implant** |
| --- | --- | --- |
| Braingate | 16 | iMEA |
| Synchron | 10 (of which 6 unpublished) | Endovascular |
| Pittsburgh/Chicago | 9 (of which 1 unpublished) | iMEA |
| Caltech | 6 (of which 2 unpublished) | iMEA |
| Neural Signals Inc/Georgia | 6 | NT |
| University of Toronto | 4 | ECoG |
| Utrecht | 4 (of which 2 unpublished) | ECoG |
| University of California San Francisco | 3 (of which 1 unpublished) | ECoG |
| Neuralink | 3 (all unpublished) | iMEA |
| Grenoble | 2 | ECoG |
| Johns Hopkins | 2 | iMEA & ECoG |
| EPFL/Lausanne | 4 (of which 3 unpublished) | ECoG |
| Tsinghua | 2 (all unpublished) | iMEA |
| Case Western Reserve University | 1 | iMEA |
| Ohio State/Battelle | 1 | iMEA |
| TJU Philadelphia | 1 | iMEA |
| University of Miami | 1 | ECoG |
| Wyss Center | 1 | iMEA |
| Zheijiang University | 1 | iMEA |
| Feinstein (Northwell Health) | 1 (unpublished) | iMEA |
| Technical University of Munich | 1 (unpublished) | iMEA |
| TECNALIA | 1 (unpublished) | iMEA |

**Supplementary Materials F: Table of studies reporting on communication speed**

| **Authors** | **Title** | **Approach** | **Reported speed value (averaged across participants)** | **Speed in WPM** | **Accuracy (averaged across participants)** |
| --- | --- | --- | --- | --- | --- |
| Bacher et al. (2015) | Neural Point-and-Click Communication by a Person With Incomplete Locked-In Syndrome. | Cursor-based typing | 9.4 CCPM | 1.88 | 92.3 +- 11.9% |
| Gilja et al. (2015) | Clinical translation of a high-performance neural prosthesis. | Cursor-based typing | 6 WPM | 6 | N/A |
| Jarosiewicz et al. (2015) | Virtual typing by people with tetraplegia using a self-calibrating intracortical brain-computer interface. | Cursor-based typing | 12 CCPM | 2.4 | N/A |
| Kennedy et al. (2000) | Direct control of a computer from the human central nervous system. | Cursor-based typing | 3 CPM | 0.6 | N/A |
| Luo et al. (2023) | Stable Decoding from a Speech BCI Enables Control for an Individual with ALS without Recalibration for 3 Months. | Speech decoding | 14.9 WPM | 14.9 | 90.59% |
| Metzger et al. (2022) | Generalizable spelling using a speech neuroprosthesis in an individual with severe limb and vocal paralysis | Speech decoding | 8.6 WPM | 8.6 | 93.87% |
| Mitchell et al. (2023) | Assessment of Safety of a Fully Implanted Endovascular Brain-Computer Interface for Severe Paralysis in 4 Patients: The Stentrode With Thought-Controlled Digital Switch (SWITCH) Study. | Cursor-based typing | 16.6 CCPM | 3.32 | 93.9% |
| Oxley et al. (2020) | Motor neuroprosthesis implanted using cerebral venography improves activities of daily living in severe paralysis | Cursor-based typing | 16.95 CCPM | 3.39 | 92.9% |
| Pandarinath et al. (2017) | High performance communication by people with paralysis using an intracortical brain-computer interface | Cursor-based typing | 27.7 CCPM | 5.54 | N/A |
| Metzger et al. (2023) | A high-performance neuroprosthesis for speech decoding and avatar control | Speech decoding | 78 WPM | 78 | 75% |
| Nuyujukian et al. (2018) | Cortical control of a tablet computer by people with paralysis | Cursor-based typing | 22.8 CCPM | 4.56 | N/A |
| Willett et al. (2021) | High-performance brain-to-text communication via handwriting | Other | 90 CPM | 16.938 | 94.1% |
| Moses et al. (2021) | Neuroprosthesis for Decoding Speech in a Paralyzed Person with Anarthria | Speech decoding | 15.2 WPM | 15.2 | 74.4% |
| Willett et al. (2023) | A high-performance speech neuroprosthesis | Speech decoding | 62 WPM | 62 | 76.2% |
| Candrea et al. (2024) | A click-based electrocorticographic brain-computer interface enables long-term high-performance switch scan spelling. | Cursor-based typing | 10.2 CCPM | 2.04 | 93.6% |
| Card et al. (2024) | An accurate and rapidly calibrating speech neuroprosthesis. | Speech decoding | 31.6 WPM | 31.6 | 90.2% |
| Fan et al. (2023) | Plug-and-Play Stability for Intracortical Brain-Computer Interfaces: A One-Year Demonstration of Seamless Brain-to-Text Communication. | Other | 14.6 WPM | 14.6 | 93.84% |

**References**

Aflalo, T., Kellis, S., Klaes, C., Lee, B., Shi, Y., Pejsa, K., et al. (2015). Decoding motor imagery from the posterior parietal cortex of a tetraplegic human. *Science* 348, 906–910. doi: 10.1126/science.aaa5417

Ajiboye, A. B., Simeral, J. D., Donoghue, J. P., Hochberg, L. R., and Kirsch, R. F. (2012). Prediction of imagined single-joint movements in a person with high-level tetraplegia. *IEEE Trans. Biomed. Eng.* 59, 2755–2765. doi: 10.1109/TBME.2012.2209882

Ajiboye, A. B., Willett, F. R., Young, D. R., Memberg, W. D., Murphy, B. A., Miller, J. P., et al. (2017). Restoration of reaching and grasping in a person with tetraplegia through brain-controlled muscle stimulation: a proof-of-concept demonstration. *Lancet Lond. Engl.* 389, 1821. doi: 10.1016/S0140-6736(17)30601-3

Angrick, M., Luo, S., Rabbani, Q., Candrea, D. N., Shah, S., Milsap, G. W., et al. (2024). Online speech synthesis using a chronically implanted brain–computer interface in an individual with ALS. *Sci. Rep.* 14, 9617. doi: 10.1038/s41598-024-60277-2

Bacher, D., Jarosiewicz, B., Masse, N. Y., Stavisky, S. D., Simeral, J. D., Newell, K., et al. (2015). Neural Point-and-Click Communication by a Person With Incomplete Locked-In Syndrome. *Neurorehabil. Neural Repair* 29, 462–471. doi: 10.1177/1545968314554624

Bashford, L., Rosenthal, I. A., Kellis, S., Bjånes, D., Pejsa, K., Brunton, B. W., et al. (2024). Neural subspaces of imagined movements in parietal cortex remain stable over several years in humans. *J. Neural Eng.* 21, 046059. doi: 10.1088/1741-2552/ad6e19

Benabid, A. L., Costecalde, T., Eliseyev, A., Charvet, G., Verney, A., Karakas, S., et al. (2019). An exoskeleton controlled by an epidural wireless brain–machine interface in a tetraplegic patient: a proof-of-concept demonstration. *Lancet Neurol.* 18, 1112–1122. doi: 10.1016/S1474-4422(19)30321-7

Bockbrader, M., Annetta, N., Friedenberg, D., Schwemmer, M., Skomrock, N., Colachis, S., et al. (2019). Clinically Significant Gains in Skillful Grasp Coordination by an Individual With Tetraplegia Using an Implanted Brain-Computer Interface With Forearm Transcutaneous Muscle Stimulation. *Arch. Phys. Med. Rehabil.* 100, 1201–1217. doi: 10.1016/j.apmr.2018.07.445

Bouton, C. E., Shaikhouni, A., Annetta, N. V., Bockbrader, M. A., Friedenberg, D. A., Nielson, D. M., et al. (2016). Restoring cortical control of functional movement in a human with quadriplegia. *Nature* 533, 247–250. doi: 10.1038/nature17435

Brandman, D. M., Burkhart, M. C., Kelemen, J., Franco, B., Harrison, M. T., and Hochberg, L. R. (2018a). Robust Closed-Loop Control of a Cursor in a Person with Tetraplegia using Gaussian Process Regression. *Neural Comput.*, 1–23. doi: 10.1162/neco_a_01129

Brandman, D. M., Hosman, T., Saab, J., Burkhart, M. C., Shanahan, B. E., Ciancibello, J. G., et al. (2018b). Rapid calibration of an intracortical brain computer interface for people with tetraplegia. *J. Neural Eng.* 15, 026007. doi: 10.1088/1741-2552/aa9ee7

Brumberg, J. S., Wright, E. J., Andreasen, D. S., Guenther, F. H., and Kennedy, P. R. (2011). Classification of intended phoneme production from chronic intracortical microelectrode recordings in speech motor cortex. *Front. Neurosci.* 5. doi: 10.3389/fnins.2011.00065

Cajigas, I., Davis, K. C., Meschede-Krasa, B., Prins, N. W., Gallo, S., Naeem, J. A., et al. (2021). Implantable brain–computer interface for neuroprosthetic-enabled volitional hand grasp restoration in spinal cord injury. *Brain Commun.* 3, fcab248. doi: 10.1093/braincomms/fcab248

Cajigas, I., Davis, K. C., Prins, N. W., Gallo, S., Naeem, J. A., Fisher, L., et al. (2023). Brain-Computer interface control of stepping from invasive electrocorticography upper-limb motor imagery in a patient with quadriplegia. *Front. Hum. Neurosci.* 16, 1077416. doi: 10.3389/fnhum.2022.1077416

Candrea, D. N., Shah, S., Luo, S., Angrick, M., Rabbani, Q., Coogan, C., et al. (2024). A click-based electrocorticographic brain-computer interface enables long-term high-performance switch scan spelling. *Commun. Med.* 4, 1–14. doi: 10.1038/s43856-024-00635-3

Card, N. S., Wairagkar, M., Iacobacci, C., Hou, X., Singer-Clark, T., Willett, F. R., et al. (2024). An accurate and rapidly calibrating speech neuroprosthesis. *medRxiv*, 2023.12.26.23300110. doi: 10.1101/2023.12.26.23300110

Chadwick, E. K., Blana, D., Simeral, J. D., Lambrecht, J., Kim, S. P., Cornwell, A. S., et al. (2011). Continuous neuronal ensemble control of simulated arm reaching by a human with tetraplegia. *J. Neural Eng.* 8, 034003. doi: 10.1088/1741-2560/8/3/034003

Chaudhary, U., Vlachos, I., Zimmermann, J. B., Espinosa, A., Tonin, A., Jaramillo-Gonzalez, A., et al. (2022). Spelling interface using intracortical signals in a completely locked-in patient enabled via auditory neurofeedback training. *Nat. Commun.* 13, 1236. doi: 10.1038/s41467-022-28859-8

Colachis, S. C., Bockbrader, M. A., Zhang, M., Friedenberg, D. A., Annetta, N. V., Schwemmer, M. A., et al. (2018). Dexterous Control of Seven Functional Hand Movements Using Cortically-Controlled Transcutaneous Muscle Stimulation in a Person With Tetraplegia. *Front. Neurosci.* 12. doi: 10.3389/fnins.2018.00208

Colachis, S. C., Dunlap, C. F., Annetta, N. V., Tamrakar, S. M., Bockbrader, M. A., and Friedenberg, D. A. (2021). Long-term intracortical microelectrode array performance in a human: a 5 year retrospective analysis. *J. Neural Eng.* 18, 0460d7. doi: 10.1088/1741-2552/ac1add

Collinger, J. L., Wodlinger, B., Downey, J. E., Wang, W., Tyler-Kabara, E. C., Weber, D. J., et al. (2013). High-performance neuroprosthetic control by an individual with tetraplegia. *The Lancet* 381, 557–564. doi: 10.1016/S0140-6736(12)61816-9

Davis, K. C., Meschede-Krasa, B., Cajigas, I., Prins, N. W., Alver, C., Gallo, S., et al. (2022). Design-development of an at-home modular brain–computer interface (BCI) platform in a case study of cervical spinal cord injury. *J. NeuroEngineering Rehabil.* 19, 53. doi: 10.1186/s12984-022-01026-2

Degenhart, A. D., Hiremath, S. V., Yang, Y., Foldes, S., Collinger, J. L., Boninger, M., et al. (2018). Remapping cortical modulation for electrocorticographic brain-computer interfaces: a somatotopy-based approach in individuals with upper-limb paralysis. *J. Neural Eng.* 15, 026021. doi: 10.1088/1741-2552/aa9bfb

Dekleva, B. M., Chowdhury, R. H., Batista, A. P., Chase, S. M., Yu, B. M., Boninger, M. L., et al. (2024). Motor cortex retains and reorients neural dynamics during motor imagery. *Nat. Hum. Behav.* 8, 729–742. doi: 10.1038/s41562-023-01804-5

Dekleva, B. M., Weiss, J. M., Boninger, M. L., and Collinger, J. L. (2021). Generalizable cursor click decoding using grasp-related neural transients. *J. Neural Eng.* 18, 10.1088/1741-2552/ac16b2. doi: 10.1088/1741-2552/ac16b2

Deo, D. R., Rezaii, P., Hochberg, L. R., M Okamura, A., Shenoy, K. V., and Henderson, J. M. (2021). Effects of Peripheral Haptic Feedback on Intracortical Brain-Computer Interface Control and Associated Sensory Responses in Motor Cortex. *IEEE Trans. Haptics* 14, 762–775. doi: 10.1109/TOH.2021.3072615

Downey, J. E., Brane, L., Gaunt, R. A., Tyler-Kabara, E. C., Boninger, M. L., and Collinger, J. L. (2017). Motor cortical activity changes during neuroprosthetic-controlled object interaction. *Sci. Rep.* 7, 16947. doi: 10.1038/s41598-017-17222-3

Downey, J. E., Weiss, J. M., Flesher, S. N., Thumser, Z. C., Marasco, P. D., Boninger, M. L., et al. (2018). Implicit Grasp Force Representation in Human Motor Cortical Recordings. *Front. Neurosci.* 12, 801. doi: 10.3389/fnins.2018.00801

Downey, J. E., Weiss, J. M., Muelling, K., Venkatraman, A., Valois, J.-S., Hebert, M., et al. (2016). Blending of brain-machine interface and vision-guided autonomous robotics improves neuroprosthetic arm performance during grasping. *J. NeuroEngineering Rehabil.* 13, 28. doi: 10.1186/s12984-016-0134-9

Fan, C., Hahn, N., Kamdar, F., Avansino, D., Wilson, G. H., Hochberg, L., et al. (2023). Plug-and-Play Stability for Intracortical Brain-Computer Interfaces: A One-Year Demonstration of Seamless Brain-to-Text Communication. *Adv. Neural Inf. Process. Syst.* 36, 42258–42270.

Feng, Z., Sun, Y., Qian, L., Qi, Y., Wang, Y., Guan, C., et al. (2022). Design a Novel BCI for Neurorehabilitation Using Concurrent LFP and EEG Features: A Case Study. *IEEE Trans. Biomed. Eng.* 69, 1554–1563. doi: 10.1109/TBME.2021.3115799

Flesher, S. N., Downey, J. E., Weiss, J. M., Hughes, C. L., Herrera, A. J., Tyler-Kabara, E. C., et al. (2021). A brain-computer interface that evokes tactile sensations improves robotic arm control. *Science* 372, 831–836. doi: 10.1126/science.abd0380

Freudenburg, Z. V., Branco, M. P., Leinders, S., van der Vijgh, B. H., Pels, E. G. M., Denison, T., et al. (2019). Sensorimotor ECoG Signal Features for BCI Control: A Comparison Between People With Locked-In Syndrome and Able-Bodied Controls. *Front. Neurosci.* 13, 1058. doi: 10.3389/fnins.2019.01058

Friedenberg, D. A., Schwemmer, M. A., Landgraf, A. J., Annetta, N. V., Bockbrader, M. A., Bouton, C. E., et al. (2017). Neuroprosthetic-enabled control of graded arm muscle contraction in a paralyzed human. *Sci. Rep.* 7, 8386. doi: 10.1038/s41598-017-08120-9

Ganesh, A., Cervantes, A. J., and Kennedy, P. R. (2022). Slow Firing Single Units Are Essential for Optimal Decoding of Silent Speech. *Front. Hum. Neurosci.* 16, 874199. doi: 10.3389/fnhum.2022.874199

Gilja, V., Pandarinath, C., Blabe, C. H., Nuyujukian, P., Simeral, J. D., Sarma, A. A., et al. (2015). Clinical translation of a high-performance neural prosthesis. *Nat. Med.* 21, 1142–1145. doi: 10.1038/nm.3953

Guan, C., Aflalo, T., Kadlec, K., Gámez de Leon, J., Rosario, E. R., Bari, A., et al. (2023). Decoding and geometry of ten finger movements in human posterior parietal cortex and motor cortex. *J. Neural Eng.* 20, 036020. doi: 10.1088/1741-2552/acd3b1

Guan, C., Aflalo, T., Zhang, C. Y., Amoruso, E., Rosario, E. R., Pouratian, N., et al. (2022). Stability of motor representations after paralysis. *eLife* 11, e74478. doi: 10.7554/eLife.74478

Guenther, F. H., Brumberg, J. S., Wright, E. J., Nieto-Castanon, A., Tourville, J. A., Panko, M., et al. (2009). A Wireless Brain-Machine Interface for Real-Time Speech Synthesis. *PLoS ONE* 4, e8218. doi: 10.1371/journal.pone.0008218

Guthrie, M. D., Herrera, A. J., Downey, J. E., Brane, L. J., Boninger, M. L., and Collinger, J. L. (2022). The impact of distractions on intracortical brain–computer interface control of a robotic arm. *Brain-Comput. Interfaces* 9, 23–35. doi: 10.1080/2326263X.2021.1980292

Handelman, D. A., Osborn, L. E., Thomas, T. M., Badger, A. R., Thompson, M., Nickl, R. W., et al. (2022). Shared Control of Bimanual Robotic Limbs With a Brain-Machine Interface for Self-Feeding. *Front. Neurorobotics* 16. doi: 10.3389/fnbot.2022.918001

Herring, E. Z., Graczyk, E. L., Memberg, W. D., Adams, R., Fernandez Baca-Vaca, G., Hutchison, B. C., et al. (2024). Reconnecting the Hand and Arm to the Brain: Efficacy of Neural Interfaces for Sensorimotor Restoration After Tetraplegia. *Neurosurgery* 94, 864–874. doi: 10.1227/neu.0000000000002769

Hochberg, L. R., Bacher, D., Jarosiewicz, B., Masse, N. Y., Simeral, J. D., Vogel, J., et al. (2012). Reach and grasp by people with tetraplegia using a neurally controlled robotic arm. *Nature* 485, 372–375. doi: 10.1038/nature11076

Hochberg, L. R., Serruya, M. D., Friehs, G. M., Mukand, J. A., Saleh, M., Caplan, A. H., et al. (2006). Neuronal ensemble control of prosthetic devices by a human with tetraplegia. *Nature* 442, 164–171. doi: 10.1038/nature04970

Jarosiewicz, B., Masse, N. Y., Bacher, D., Cash, S. S., Eskandar, E., Friehs, G., et al. (2013). Advantages of closed-loop calibration in intracortical brain-computer interfaces for people with tetraplegia. *J. Neural Eng.* 10, 046012. doi: 10.1088/1741-2560/10/4/046012

Jarosiewicz, B., Sarma, A. A., Bacher, D., Masse, N. Y., Simeral, J. D., Sorice, B., et al. (2015). Virtual typing by people with tetraplegia using a self-calibrating intracortical brain-computer interface. *Sci. Transl. Med.* 7, 313ra179. doi: 10.1126/scitranslmed.aac7328

Jarosiewicz, B., Sarma, A. A., Saab, J., Franco, B., Cash, S. S., Eskandar, E. N., et al. (2016). Retrospectively supervised click decoder calibration for self-calibrating point-and-click brain-computer interfaces. *J. Physiol. Paris* 110, 382–391. doi: 10.1016/j.jphysparis.2017.03.001

Jiang, H., Wang, R., Zheng, Z., Zhu, J., Qi, Y., Xu, K., et al. (2022). Short report: surgery for implantable brain-computer interface assisted by robotic navigation system. *Acta Neurochir. (Wien)* 164, 2299–2302. doi: 10.1007/s00701-022-05235-5

Jorge, A., Royston, D. A., Tyler-Kabara, E. C., Boninger, M. L., and Collinger, J. L. (2020). Classification of Individual Finger Movements Using Intracortical Recordings in Human Motor Cortex. *Neurosurgery* 87, 630. doi: 10.1093/neuros/nyaa026

Kennedy, P., Andreasen, D., Bartels, J., Ehirim, P., Mao, H., Velliste, M., et al. (2011). Making the lifetime connection between brain and machine for restoring and enhancing function. *Prog. Brain Res.* 194, 1–25. doi: 10.1016/B978-0-444-53815-4.00020-0

Kennedy, P. R., Bakay, R. A. E., Moore, M. M., Adams, K., and Goldwaithe, J. (2000). Direct control of a computer from the human central nervous system. *IEEE Trans. Rehabil. Eng.* 8, 198–202. doi: 10.1109/86.847815

Kennedy, P. R., Kirby, M. T., Moore, M. M., King, B., and Mallory, A. (2004). Computer control using human intracortical local field potentials. *IEEE Trans. Neural Syst. Rehabil. Eng.* 12, 339–344. doi: 10.1109/TNSRE.2004.834629

Kim, S.-P., Simeral, J. D., Hochberg, L. R., Donoghue, J. P., and Black, M. J. (2008). Neural control of computer cursor velocity by decoding motor cortical spiking activity in humans with tetraplegia. *J. Neural Eng.* 5, 455–476. doi: 10.1088/1741-2560/5/4/010

Kim, S.-P., Simeral, J. D., Hochberg, L. R., Donoghue, J. P., Friehs, G. M., and Black, M. J. (2011). Point-and-Click Cursor Control With an Intracortical Neural Interface System by Humans With Tetraplegia. *Ieee Trans. Neural Syst. Rehabil. Eng.* 19, 193–203. doi: 10.1109/TNSRE.2011.2107750

Kryger, M., Wester, B., Pohlmeyer, E. A., Rich, M., John, B., Beaty, J., et al. (2017). Flight simulation using a Brain-Computer Interface: A pilot, pilot study. *Exp. Neurol.* 287, 473–478. doi: 10.1016/j.expneurol.2016.05.013

Larzabal, C., Bonnet, S., Costecalde, T., Auboiroux, V., Charvet, G., Chabardes, S., et al. (2021). Long-term stability of the chronic epidural wireless recorder WIMAGINE in tetraplegic patients. *J. Neural Eng.* 18, 056026. doi: 10.1088/1741-2552/ac2003

Leinders, S., Vansteensel, M. J., Branco, M. P., Freudenburg, Z. V., Pels, E. G. M., Van der Vijgh, B., et al. (2020). Dorsolateral prefrontal cortex-based control with an implanted brain–computer interface. *Sci. Rep.* 10, 15448. doi: 10.1038/s41598-020-71774-5

Lorach, H., Galvez, A., Spagnolo, V., Martel, F., Karakas, S., Intering, N., et al. (2023). Walking naturally after spinal cord injury using a brain–spine interface. *Nature* 618, 126–133. doi: 10.1038/s41586-023-06094-5

Luo, S., Angrick, M., Coogan, C., Candrea, D. N., Wyse-Sookoo, K., Shah, S., et al. (2023). Stable Decoding from a Speech BCI Enables Control for an Individual with ALS without Recalibration for 3 Months. *Adv. Sci.* 10, 2304853. doi: 10.1002/advs.202304853

Márquez-Chin, C., Popovic, M. R., Sanin, E., Chen, R., and Lozano, A. M. (2012). Real-time two-dimensional asynchronous control of a computer cursor with a single subdural electrode. *J. Spinal Cord Med.* 35, 382–391. doi: 10.1179/2045772312Y.0000000043

Masse, N. Y., Jarosiewicz, B., Simeral, J. D., Bacher, D., Stavisky, S. D., Cash, S. S., et al. (2014). Non-causal spike filtering improves decoding of movement intention for intracortical BCIs. *J. Neurosci. Methods* 236, 58–67. doi: 10.1016/j.jneumeth.2014.08.004

Metzger, S. L., Littlejohn, K. T., Silva, A. B., Moses, D. A., Seaton, M. P., Wang, R., et al. (2023). A high-performance neuroprosthesis for speech decoding and avatar control. *Nature* 620, 1037–1046. doi: 10.1038/s41586-023-06443-4

Metzger, S. L., Liu, J. R., Moses, D. A., Dougherty, M. E., Seaton, M. P., Littlejohn, K. T., et al. (2022). Generalizable spelling using a speech neuroprosthesis in an individual with severe limb and vocal paralysis. *Nat. Commun.* 13, 6510. doi: 10.1038/s41467-022-33611-3

Milekovic, T., Bacher, D., Sarma, A. A., Simeral, J. D., Saab, J., Pandarinath, C., et al. (2019). Volitional control of single-electrode high gamma local field potentials by people with paralysis. *J. Neurophysiol.* 121, 1428–1450. doi: 10.1152/jn.00131.2018

Milekovic, T., Sarma, A. A., Bacher, D., Simeral, J. D., Saab, J., Pandarinath, C., et al. (2018). Stable long-term BCI-enabled communication in ALS and locked-in syndrome using LFP signals. *J. Neurophysiol.* 120, 343–360. doi: 10.1152/jn.00493.2017

Mitchell, P., Lee, S. C. M., Yoo, P. E., Morokoff, A., Sharma, R. P., Williams, D. L., et al. (2023). Assessment of Safety of a Fully Implanted Endovascular Brain-Computer Interface for Severe Paralysis in 4 Patients: The Stentrode With Thought-Controlled Digital Switch (SWITCH) Study. *JAMA Neurol.* 80, 270. doi: 10.1001/jamaneurol.2022.4847

Moly, A., Costecalde, T., Martel, F., Martin, M., Larzabal, C., Karakas, S., et al. (2022). An adaptive closed-loop ECoG decoder for long-term and stable bimanual control of an exoskeleton by a tetraplegic. *J. Neural Eng.* 19, 026021. doi: 10.1088/1741-2552/ac59a0

Moses, D. A., Metzger, S. L., Liu, J. R., Anumanchipalli, G. K., Makin, J. G., Sun, P. F., et al. (2021). Neuroprosthesis for Decoding Speech in a Paralyzed Person with Anarthria. *N. Engl. J. Med.* 385, 217–227. doi: 10.1056/NEJMoa2027540

Nickl, R. W., Anaya, M. A., Thomas, T. M., Fifer, M. S., Candrea, D. N., McMullen, D. P., et al. (2022). Characteristics and stability of sensorimotor activity driven by isolated-muscle group activation in a human with tetraplegia. *Sci. Rep.* 12, 10353. doi: 10.1038/s41598-022-13436-2

Nuyujukian, P., Albites Sanabria, J., Saab, J., Pandarinath, C., Jarosiewicz, B., Blabe, C. H., et al. (2018). Cortical control of a tablet computer by people with paralysis. *PLoS ONE* 13, e0204566. doi: 10.1371/journal.pone.0204566

Oxley, T. J., Yoo, P. E., Rind, G. S., Ronayne, S. M., Lee, C. M. S., Bird, C., et al. (2021). Motor neuroprosthesis implanted with neurointerventional surgery improves capacity for activities of daily living tasks in severe paralysis: first in-human experience. *J. Neurointerventional Surg.* 13, 102–108. doi: 10.1136/neurintsurg-2020-016862

Pandarinath, C., Gilja, V., Blabe, C. H., Nuyujukian, P., Sarma, A. A., Sorice, B. L., et al. (2015). Neural population dynamics in human motor cortex during movements in people with ALS. *eLife* 4, e07436. doi: 10.7554/eLife.07436

Pandarinath, C., Nuyujukian, P., Blabe, C. H., Sorice, B. L., Saab, J., Willett, F. R., et al. (2017). High performance communication by people with paralysis using an intracortical brain-computer interface. *eLife* 6, e18554. doi: 10.7554/eLife.18554

Pels, E. G. M., Aarnoutse, E. J., Leinders, S., Freudenburg, Z. V., Branco, M. P., van der Vijgh, B. H., et al. (2019). Stability of a chronic implanted brain-computer interface in late-stage amyotrophic lateral sclerosis. *Clin. Neurophysiol.* 130, 1798–1803. doi: 10.1016/j.clinph.2019.07.020

Perge, J. A., Homer, M. L., Malik, W. Q., Cash, S., Eskandar, E., Friehs, G., et al. (2013). Intra-day signal instabilities affect decoding performance in an intracortical neural interface system. *J. Neural Eng.* 10, 036004. doi: 10.1088/1741-2560/10/3/036004

Perge, J. A., Zhang, S., Malik, W. Q., Homer, M. L., Cash, S., Friehs, G., et al. (2014). Reliability of directional information in unsorted spikes and local field potentials recorded in human motor cortex. *J. Neural Eng.* 11, 046007. doi: 10.1088/1741-2560/11/4/046007

Rastogi, A., Vargas-Irwin, C. E., Willett, F. R., Abreu, J., Crowder, D. C., Murphy, B. A., et al. (2020). Neural Representation of Observed, Imagined, and Attempted Grasping Force in Motor Cortex of Individuals with Chronic Tetraplegia. *Sci. Rep.* 10, 1429. doi: 10.1038/s41598-020-58097-1

Rastogi, A., Willett, F. R., Abreu, J., Crowder, D. C., Murphy, B. A., Memberg, W. D., et al. (2021). The Neural Representation of Force across Grasp Types in Motor Cortex of Humans with Tetraplegia. *eNeuro* 8, ENEURO.0231-20.2020. doi: 10.1523/ENEURO.0231-20.2020

Rizzoglio, F., Altan, E., Ma, X., Bodkin, K. L., Dekleva, B. M., Solla, S. A., et al. (2023). From monkeys to humans: observation-based EMG brain–computer interface decoders for humans with paralysis. *J. Neural Eng.* 20, 056040. doi: 10.1088/1741-2552/ad038e

Rouanne, V., Costecalde, T., Benabid, A. L., and Aksenova, T. (2022). Unsupervised adaptation of an ECoG based brain–computer interface using neural correlates of task performance. *Sci. Rep.* 12, 21316. doi: 10.1038/s41598-022-25049-w

Rubin, D. B., Ajiboye, A. B., Barefoot, L., Bowker, M., Cash, S. S., Chen, D., et al. (2023). Interim Safety Profile From the Feasibility Study of the BrainGate Neural Interface System. *Neurology* 100, e1177–e1192. doi: 10.1212/WNL.0000000000201707

Rubin, D. B., Hosman, T., Kelemen, J. N., Kapitonava, A., Willett, F. R., Coughlin, B. F., et al. (2022). Learned Motor Patterns Are Replayed in Human Motor Cortex during Sleep. *J. Neurosci.* 42, 5007–5020. doi: 10.1523/JNEUROSCI.2074-21.2022

Sakellaridi, S., Christopoulos, V. N., Aflalo, T., Pejsa, K. W., Rosario, E. R., Ouellette, D., et al. (2019). Intrinsic variable learning for brain-machine interface control by human anterior intraparietal cortex. *Neuron* 102, 694-705.e3. doi: 10.1016/j.neuron.2019.02.012

Serino, A., Bockbrader, M., Bertoni, T., Colachis IV, S., Solcà, M., Dunlap, C., et al. (2022). Sense of agency for intracortical brain–machine interfaces. *Nat. Hum. Behav.* 6, 565–578. doi: 10.1038/s41562-021-01233-2

Serruya, M. D., Napoli, A., Satterthwaite, N., Kardine, J., McCoy, J., Grampurohit, N., et al. (2022). Neuromotor prosthetic to treat stroke-related paresis: N-of-1 trial. *Commun. Med.* 2, 1–14. doi: 10.1038/s43856-022-00105-8

Shah, N. P., Willsey, M. S., Hahn, N., Kamdar, F., Avansino, D. T., Hochberg, L. R., et al. (2023). A brain-computer typing interface using finger movements. *Int. IEEEEMBS Conf. Neural Eng. Proc. Int. IEEE EMBS Conf. Neural Eng.* 2023, 10.1109/ner52421.2023.10123912. doi: 10.1109/ner52421.2023.10123912

Shaikhouni, A., Donoghue, J. P., and Hochberg, L. R. (2013). Somatosensory responses in a human motor cortex. *J. Neurophysiol.* 109, 2192–2204. doi: 10.1152/jn.00368.2012

Sharma, G., Friedenberg, D. A., Annetta, N., Glenn, B., Bockbrader, M., Majstorovic, C., et al. (2016). Using an Artificial Neural Bypass to Restore Cortical Control of Rhythmic Movements in a Human with Quadriplegia. *Sci. Rep.* 6, 33807. doi: 10.1038/srep33807

Silversmith, D. B., Abiri, R., Hardy, N. F., Natraj, N., Tu-Chan, A., Chang, E. F., et al. (2021). Plug-and-play control of a brain–computer interface through neural map stabilization. *Nat. Biotechnol.* 39, 326–335. doi: 10.1038/s41587-020-0662-5

Simeral, J. D., Hosman, T., Saab, J., Flesher, S. N., Vilela, M., Franco, B., et al. (2021). Home Use of a Percutaneous Wireless Intracortical Brain-Computer Interface by Individuals With Tetraplegia. *IEEE Trans. Biomed. Eng.* 68, 2313–2325. doi: 10.1109/TBME.2021.3069119

Simeral, J. D., Kim, S.-P., Black, M. J., Donoghue, J. P., and Hochberg, L. R. (2011). Neural control of cursor trajectory and click by a human with tetraplegia 1000 days after implant of an intracortical microelectrode array. *J. Neural Eng.* 8, 025027. doi: 10.1088/1741-2560/8/2/025027

Śliwowski, M., Martin, M., Souloumiac, A., Blanchart, P., and Aksenova, T. (2022). Decoding ECoG signal into 3D hand translation using deep learning. *J. Neural Eng.* 19, 026023. doi: 10.1088/1741-2552/ac5d69

Stavisky, S. D., Willett, F. R., Avansino, D. T., Hochberg, L. R., Shenoy, K. V., and Henderson, J. M. (2020). Speech-related dorsal motor cortex activity does not interfere with iBCI cursor control. *J. Neural Eng.* 17, 016049. doi: 10.1088/1741-2552/ab5b72

Stavisky, S. D., Willett, F. R., Wilson, G. H., Murphy, B. A., Rezaii, P., Avansino, D. T., et al. (2019). Neural ensemble dynamics in dorsal motor cortex during speech in people with paralysis. *eLife* 8, e46015. doi: 10.7554/eLife.46015

Talakoub, O., Marquez-Chin, C., Popovic, M. R., Navarro, J., Fonoff, E. T., Hamani, C., et al. (2017). Reconstruction of reaching movement trajectories using electrocorticographic signals in humans. *PLoS ONE* 12, e0182542. doi: 10.1371/journal.pone.0182542

Truccolo, W., Friehs, G. M., Donoghue, J. P., and Hochberg, L. R. (2008). Primary Motor Cortex Tuning to Intended Movement Kinematics in Humans with Tetraplegia. *J. Neurosci.* 28, 1163–1178. doi: 10.1523/JNEUROSCI.4415-07.2008

Vansteensel, M. J., Leinders, S., Branco, M. P., Crone, N. E., Denison, T., Freudenburg, Z. V., et al. (2024). Longevity of a Brain-Computer Interface for Amyotrophic Lateral Sclerosis. *N. Engl. J. Med.* 391, 619–626. doi: 10.1056/NEJMoa2314598

Vansteensel, M. J., Pels, E. G. M., Bleichner, M. G., Branco, M. P., Denison, T., Freudenburg, Z. V., et al. (2016). Fully Implanted Brain–Computer Interface in a Locked-In Patient with ALS. *N. Engl. J. Med.* 375, 2060–2066. doi: 10.1056/NEJMoa1608085

Vargas-Irwin, C. E., Feldman, J. M., King, B., Simeral, J. D., Sorice, B. L., Oakley, E. M., et al. (2018). Watch, Imagine, Attempt: Motor Cortex Single-Unit Activity Reveals Context-Dependent Movement Encoding in Humans With Tetraplegia. *Front. Hum. Neurosci.* 12, 450. doi: 10.3389/fnhum.2018.00450

WANDELT, S. K., KELLIS, S., BJÅNES, D. A., PEJSA, K., LEE, B., LIU, C., et al. (2022). Decoding grasp and speech signals from the cortical grasp circuit in a tetraplegic human. *Neuron* 110, 1777-1787.e3. doi: 10.1016/j.neuron.2022.03.009

Wang, W., Collinger, J. L., Degenhart, A. D., Tyler-Kabara, E. C., Schwartz, A. B., Moran, D. W., et al. (2013). An Electrocorticographic Brain Interface in an Individual with Tetraplegia. *PLoS ONE* 8, e55344. doi: 10.1371/journal.pone.0055344

Weiss, J. M., Gaunt, R. A., Franklin, R., Boninger, M. L., and Collinger, J. L. (2019). Demonstration of a portable intracortical brain-computer interface. *Brain-Comput. Interfaces* 6, 106–117. doi: 10.1080/2326263X.2019.1709260

Willett, F. R., Avansino, D. T., Hochberg, L. R., Henderson, J. M., and Shenoy, K. V. (2021). High-performance brain-to-text communication via handwriting. *Nature* 593, 249–254. doi: 10.1038/s41586-021-03506-2

Willett, F. R., Kunz, E. M., Fan, C., Avansino, D. T., Wilson, G. H., Choi, E. Y., et al. (2023). A high-performance speech neuroprosthesis. *Nature* 620, 1031–1036. doi: 10.1038/s41586-023-06377-x

Willett, F. R., Young, D. R., Murphy, B. A., Memberg, W. D., Blabe, C. H., Pandarinath, C., et al. (2019). Principled BCI Decoder Design and Parameter Selection Using a Feedback Control Model. *Sci. Rep.* 9, 8881. doi: 10.1038/s41598-019-44166-7

Wilson, G. H., Stavisky, S. D., Willett, F. R., Avansino, D. T., Kelemen, J. N., Hochberg, L. R., et al. (2020). Decoding spoken English from intracortical electrode arrays in dorsal precentral gyrus. *J. Neural Eng.* 17, 066007. doi: 10.1088/1741-2552/abbfef

Wodlinger, B., Downey, J. E., Tyler-Kabara, E. C., Schwartz, A. B., Boninger, M. L., and Collinger, J. L. (2014). Ten-dimensional anthropomorphic arm control in a human brain−machine interface: difficulties, solutions, and limitations. *J. Neural Eng.* 12, 016011. doi: 10.1088/1741-2560/12/1/016011

Wyse-Sookoo, K., Luo, S., Candrea, D., Schippers, A., Tippett, D. C., Wester, B., et al. (2024). Stability of ECoG high gamma signals during speech and implications for a speech BCI system in an individual with ALS: a year-long longitudinal study. *J. Neural Eng.* 21, 046016. doi: 10.1088/1741-2552/ad5c02

Young, D., Willett, F., Memberg, W. D., Murphy, B., Rezaii, P., Walter, B., et al. (2019). Closed-loop cortical control of virtual reach and posture using Cartesian and joint velocity commands. *J. Neural Eng.* 16, 026011. doi: 10.1088/1741-2552/aaf606

YOUNG, D., WILLETT, F., MEMBERG, W. D., MURPHY, B., WALTER, B., SWEET, J., et al. (2018). Signal processing methods for reducing artifacts in microelectrode brain recordings caused by functional electrical stimulation. *J. Neural Eng.* 15, 026014. doi: 10.1088/1741-2552/aa9ee8

Zhang, C. Y., Aflalo, T., Revechkis, B., Rosario, E., Ouellette, D., Pouratian, N., et al. (2020). Preservation of Partially Mixed Selectivity in Human Posterior Parietal Cortex across Changes in Task Context. *eNeuro* 7, ENEURO.0222-19.2019. doi: 10.1523/ENEURO.0222-19.2019

Zhang, M., Schwemmer, M. A., Ting, J. E., Majstorovic, C. E., Friedenberg, D. A., Bockbrader, M. A., et al. (2018). Extracting wavelet based neural features from human intracortical recordings for neuroprosthetics applications. *Bioelectron. Med.* 4, 11. doi: 10.1186/s42234-018-0011-x
